# Fibrocyte enrichment and myofibroblastic adaptation causes nucleus pulposus fibrosis and associates with disc degeneration severity

**DOI:** 10.1038/s41413-024-00372-2

**Published:** 2025-01-20

**Authors:** Yi Sun, Yan Peng, Zezhuo Su, Kyle, K. H. So, Qiuji Lu, Maojiang Lyu, Jianwei Zuo, Yongcan Huang, Zhiping Guan, Kenneth M. C. Cheung, Zhaomin Zheng, Xintao Zhang, Victor Y. L. Leung

**Affiliations:** 1 Department of Sports Medicine, Peking University Shenzhen Hospital, Shenzhen, Guangdong China; 2 Department of Orthopaedics and Traumatology, The University of Hong Kong, Hong Kong SAR, China; 3 Department of Spine Surgery, Shenzhen Engineering Laboratory of Orthopaedic Regenerative Technologies, Peking University Shenzhen Hospital, Shenzhen, Guangdong China; 4 Department of Spine Surgery, the First Affiliated Hospital, Sun Yat-sen University, Guangzhou, Guangdong China

**Keywords:** Pathogenesis, Physiology

## Abstract

Fibrotic remodeling of nucleus pulposus (NP) leads to structural and mechanical anomalies of intervertebral discs that prone to degeneration, leading to low back pain incidence and disability. Emergence of fibroblastic cells in disc degeneration has been reported, yet their nature and origin remain elusive. In this study, we performed an integrative analysis of multiple single-cell RNA sequencing datasets to interrogate the cellular heterogeneity and fibroblast-like entities in degenerative human NP specimens. We found that disc degeneration severity is associated with an enrichment of fibrocyte phenotype, characterized by CD45 and collagen I dual positivity, and expression of myofibroblast marker α-smooth muscle actin. Refined clustering and classification distinguished the fibrocyte-like populations as subtypes in the NP cells - and immunocytes-clusters, expressing disc degeneration markers *HTRA1* and *ANGPTL4* and genes related to response to TGF-β. In injury-induced mouse disc degeneration model, fibrocytes were found recruited into the NP undergoing fibrosis and adopted a myofibroblast phenotype. Depleting the fibrocytes in CD11b-DTR mice in which myeloid-derived lineages were ablated by diphtheria toxin could markedly attenuate fibrous modeling and myofibroblast formation in the NP of the degenerative discs, and prevent disc height loss and histomorphological abnormalities. Marker analysis supports that disc degeneration progression is dependent on a function of CD45^+^COL1A1^+^ and αSMA^+^ cells. Our findings reveal that myeloid-derived fibrocytes play a pivotal role in NP fibrosis and may therefore be a target for modifying disc degeneration and promoting its repair.

## Introduction

The intervertebral discs (IVDs) are the largest avascular connective tissues in the body, playing a central role in spine movement. IVD degeneration (IDD) is associated with fibrosis of the nucleus pulposus (NP), the innermost colloidal core of IVDs, along with a loss of hydration and swelling pressure. The degeneration involves a degradation of hyaline extracellular matrix (ECM), in particular aggrecan and collagen II, and accumulation of fibrotic components including collagen I (COL1) and small leucin-rich proteoglycans such as biglycan and fibronectin.^1^ This leads to reduced tissue mechanical strength and hence disc prolapse under load, ultimately causing back pain and myelopathy.^2^ Previous study has shown that transplantation of mesenchymal stromal cells (MSCs)^3^ can alleviate IDD via inhibiting NP fibrosis. Understanding and controlling the profibrotic events may therefore hold the key to modifying the degeneration process. However, the mechanism of NP fibrosis and the origin of the fibroblastic population in the IDD remains elusive.

The primitive NP is initially populated by forkhead transcription factor (*FOXA2*)- or brachyury (*TBXT*)-expressing vacuolated cells derived from the notochord.^4–6^ In humans, these vacuolated cells are gradually replaced by the chondrocyte-like cells after birth.^7^ Tissue fibrosis relates to an excessive fibroblast and myofibroblast activity.^8^ Reports have shown an increased number of α-smooth muscle actin (αSMA), fibroblast-specific protein 1 (FSP1) and COL1 positive fibroblastic cells in the degenerative IVDs.^4,9^ In fact, NP cells (NPC) have a capacity to become FSP1^+^ cells when exposed to excessive TGF-β^5,10^ as well as undergo fibroblastic/myofibroblastic transition in an injury-induced IDD model.^4^ However, tracing study indicated that not all myofibroblastic cells are derived from local cells,^4^ implying alternative origins of effector cells for NP fibrosis. In line with this notion, single cell transcriptomic studies have revealed that the NP of human degenerative IVDs^7,11–14^ and animal model of IDD^6^ contain cell types other than IVD cells. These include endothelial cells, myeloid granulocytic suppressor cells, CCR2^+^ monocytes and derivatives of macrophages. Consistent with the finding, studies showed that bone marrow cells could migrate into mechanically overloaded IVD explants^15^ and that focal enrichment of fibroblastic cells could be found near the infiltrating blood vessels in the degenerative IVDs.^16^ These evidences support that access of extrinsic cells to the degenerated IVD may mediate NP fibrosis.

We hypothesized that cell sources other than resident disc cells can contribute to NP fibrosis. In this study, we interrogated the cell hierarchy in the NP of degenerative IVDs through an integrative analysis of the published single cell transcriptomes and characterization in human tissue and cells. Our findings indicated an emergence of a small hematopoietic population that possess a fibrocyte phenotype and myofibroblastic identity in the degenerative samples. Using a mouse model that labels or depletes myeloid-derived cells, we analyzed the time course of the disc fibrocytes appearance and identified their role in NP fibrosis and IDD progression. Our study implies a function of circulatory fibrocytes in NP fibrosis and as a target for modifying IDD.

## Results

### Identification of myofibroblastic NPC in IDD and NP fibrosis

**Fig. 1:**
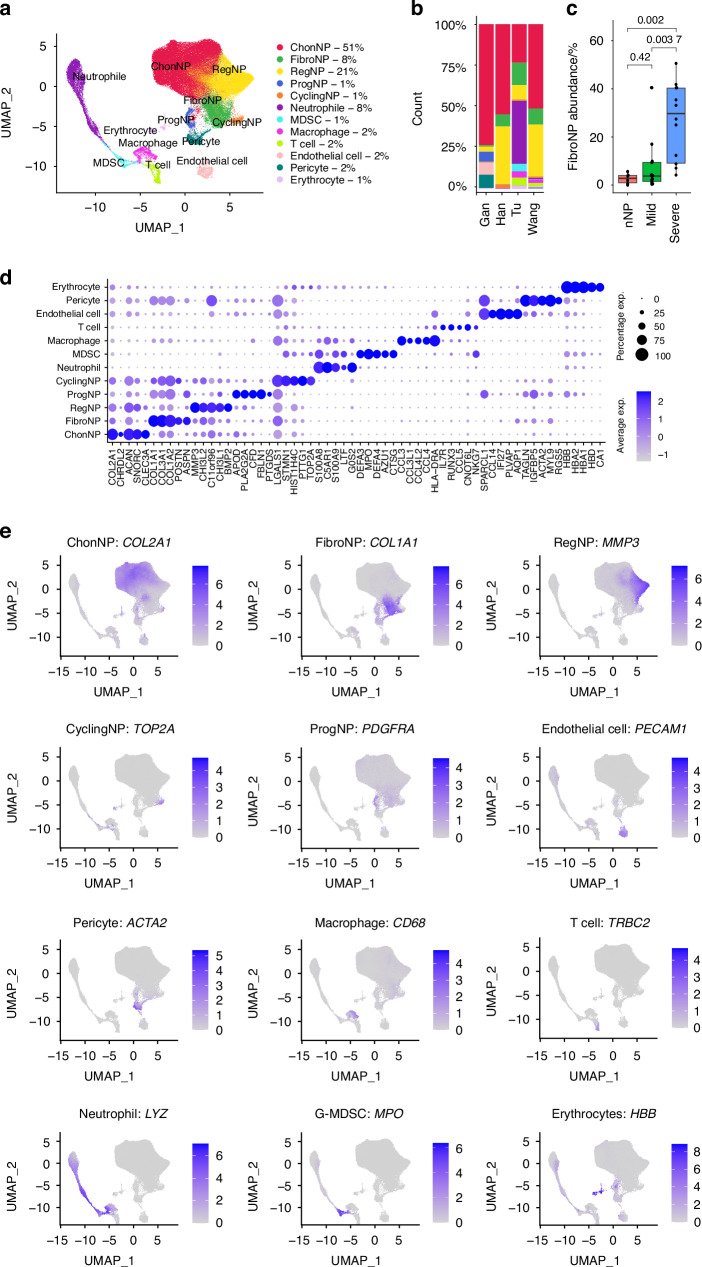
Integrative single-cell transcriptome analysis. **a** Identification of 12 distinct cell populations by unsupervised clustering from an integrative analysis of four published single cell RNA sequencing (scRNA-seq) datasets using human disc/NP tissues. **b** Distribution of the cell populations in four individual datasets. **c** Degeneration-related FibroNP abundance. nNP: non-degenerative NP (Pfirrmann I); mild and severe: mildly (Pfirrmann II–III) or severely (Pfirrmann IV-V) degenerative NP. **d** Dot plot showing the expression levels of top 5 differentially expressed genes in the cell clusters defined in (**a**). **e** Signature gene expression for the 12 cell clusters. *P* values were calculated based on unpaired t test

We examined the cellular heterogeneity and fibroblastic cell entities by an integrative analysis of 4 published single-cell RNA sequencing (scRNA-seq) data derived from cells isolated from NP tissues^7,12,14^ or recognized as NPC.^11^ The datasets consist of 6 non-degenerative human IVDs (3 scoliosis, 1 spine fracture, 1 spinal injury and 1 brain death cases), as well as 13 mildly (Pfirrmann II–III) and 12 severely (Pfirrmann IV-V) degenerative human IVDs (23 disc herniation and 2 burst fracture cases) (Table 1). A number of existing NPC phenotypes were previously identified from these datasets (marker genes summarized in Table S1). The meta-analysis identified 12 cell clusters from a total of 192 184 cells and the cell types were annotated based on their cluster differentially expressed genes (DEGs, SI file 1 and Fig. 1a, d). These clusters were mostly present across the four datasets although their proportion and distribution varied (Fig. 1b). Clustered NPC were identified by their expression of *ACAN*, *COL2A1,* and *SOX9* (SI file 1). The most abundant NPC population was the chondrogenic cluster (ChonNP) (51%) which expressed *COL2A1*, *ACAN* and *CNMD*. The regulatory cluster (RegNP) (21%) expressed *MMP3*, *GPX3* and *CHI3L2/1*. These RegNP gene markers were also expressed in the inflammatory C1/3 chonNPC and regulatory NPC reported by Han et al.^7^ and Tu et al.^12^ respectively (Table S1). The fibroblastic cluster (FibroNP; 8%) expressed *COL1A1*, *COL3A1*, *POSTN* and *ASPN*, which are known to associate with tissue fibrosis. We found that the proportion of FibroNP significantly increased with IDD severity and presented as a major NPC population (30%) in the severely degenerated samples (Fig. 1c and Fig. S1). The cycling cluster (CyclingNP) (1%) expressed the cell proliferation genes including *TOP2A* and *STMN1*, containing two distinct subsets expressing high level of either collagen I or II gene (*COL1A1*^*high*^ and *COL2A1*^*high*^). The progenitor cluster (ProgNP) (1%), distinguished by the expression of *PDGFRA* and *PLA2G2A*, was unique to the non-degenerative samples as reported by Gan et al.^14^ In addition to 5 NPC clusters, we also identified neutrophils (*LYZ*^+^), granulocyte-like myeloid-derived suppressor cells (G-MDSC^+^; *MPO*^+^), macrophages (*CD68*^+^), T cells (*TRBC2*^+^), endothelial cells (*CD34*^*+*^ and *PECAM1*^+^), pericytes (*RGS5*^*+*^ and *ACTA2*^+^), and erythrocytes (*HBB*^+^) (Fig. 1d, e). Endothelial cells (2%) were also found, which highly expressed *PECAM1* and *CD34* (Fig. 1e and Fig. S2). Erythrocytes (1%) were defined by gene expression of *HBB* (Fig. 1e).

**Table 1:** Demographic data

Data availability	Sample ID	Gender	Age	Pfirrmann grade	Diagnosis	Labeling in analysis	Refs.
GSE160756	GSM4878538	M	16	I	Spine fracture	Non-degenerative	^14^
	GSM4878539	M	31	I	Brain death	Non-degenerative	
	GSM4878540	M	31	I	Brain death	Non-degenerative	
CNP0002664	Ctrl	M	37	I	Spinal cord injury	Non-degenerative	^7^
	NP2	F	65	V	Herniation	Severe	
	NP4	M	59	III	Herniation	Mild	
	NP8	M	50	IV	Herniation	Severe	
	NP9	M	56	III	Herniation	Mild	
	NP10	F	48	III	Herniation/spondylolisthesis	Mild	
GSE165722	S1	M	63	II	Burst fracture	Mild	^12^
	S2	M	41	II	Burst fracture	Mild	
	S3	F	56	III	Herniation	Mild	
	S4	F	65	III	Herniation	Mild	
	S5	F	64	IV	Herniation	Severe	
	S6	F	53	IV	Herniation	Severe	
	S7	M	54	V	Herniation	Severe	
	S8	M	56	V	Herniation	Severe	
PRJCA014236	D1_1	M	7	I	Scoliosis	Non-degenerative	^11^
	D1_2	F	6	I	Scoliosis	Non-degenerative	
	D2_1	M	19	II	Herniation/spondylolisthesis	Mild	
	D2_2	F	46	II	Herniation/spondylolisthesis	Mild	
	D2_3	M	15	II	Herniation/spondylolisthesis	Mild	
	D3_1	M	37	III	Herniation/spondylolisthesis	Mild	
	D3_2	F	31	III	Herniation/spondylolisthesis	Mild	
	D3_3	M	30	III	Herniation/spondylolisthesis	Mild	
	D4_1	F	44	IV	Herniation/spondylolisthesis	Severe	
	D4_2	F	43	IV	Herniation/spondylolisthesis	Severe	
	D4_3	M	54	IV	Herniation/spondylolisthesis	Severe	
	D5_1	M	68	V	Herniation/spondylolisthesis	Severe	
	D5_2	F	50	V	Herniation/spondylolisthesis	Severe	
	D5_3	F	57	V	Herniation/spondylolisthesis	Severe	

A total of 31 subjects from 4 studies were selected with degeneration graded I–V, and their single cell RNA sequence data were extracted and integrated for further analysis. Samples defined as Pfirrmann grade I were considered as non-degenerative; grades II/III and IV/V as mild and severe degeneration respectively. F: female; M: male

**Fig. 2:**
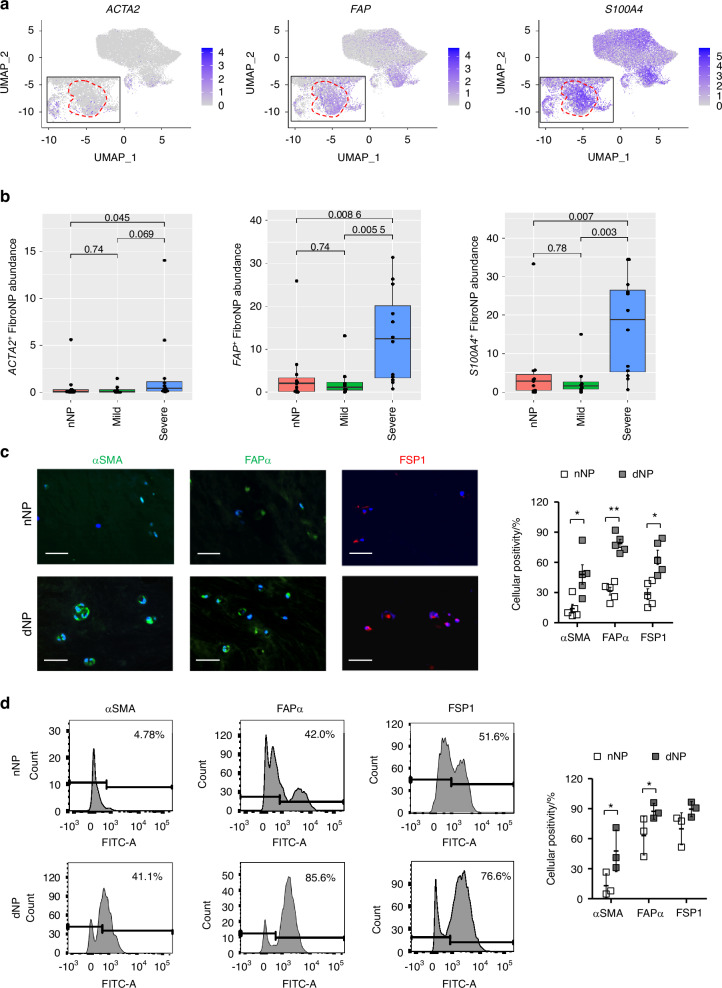
Characterization of myofibroblastic NP cells in human disc degeneration. **a** UMAP distribution of myofibroblastic markers *ACTA2* (encoding αSMA), *FAP* (encoding FAPα), and *S100A4* (encoding FSP1) in NPC, and (**b**) their expressing FibroNP abundance related to human disc degeneration severity. *P* values were calculated based on unpaired *t*-test. nNP: non-degenerative NP (Pfirrmann I); mild and severe: mildly (Pfirrmann II–III) or severely (Pfirrmann IV–V) degenerative NP. **c** Immunofluorescence and quantification of three fibroblastic marker expression in human colloidal NP tissues (*n* = 5). nNP: non-degenerative NP from scoliosis subjects; dNP: degenerative NP from degenerative disc disease subjects. Scale bars: 50 μm. Nuclei were counterstained with DAPI (Blue). **d** Representative flow cytometry plots to identify myofibroblastic NP cells (NPC). Percentage of αSMA^+^, FAPα^+^ or FSP1^+^ cells was averaged from three independent experiments. *P < 0.05, **P < 0.01 by two-tailed unpaired *t*-tests

Contractile myofibroblasts are primary effector cells of tissue fibrosis.^8^ Fibroblast activation protein-α (FAPα, encoded by *FAP*) and FSP1 (encoded by *S100A4*) mark the active fibroblasts or myofibroblast precursors,^17^ and αSMA (encoded by *ACTA2*) marks the mature myofibroblasts.^18^ These three markers, which were used to indicate fibroblastic NPC in mouse IDD,^4,9^ were found enriched in the FibroNP (Fig. 2a). A markedly higher abundance of *ACTA2*^*+*^*, FAP*^*+*^ and *S100A4*^*+*^ FibroNP cells were found in the severely degenerative NP (dNP) than the mildly degenerative or the non-degenerative NP (nNP) (Fig. 2b). More fibrillar collagens, specifically COL1 and thin collagen III, were predominantly deposited in human degenerative IVDs, which together with reduction of hyaline matrix aggrecan and collagen II, indicated NP fibrosis (Fig. S3). Immunofluorescence further verified a higher number of cells expressing αSMA (14.0% vs 48%), FAPα (31.4% vs 78.8%) and FSP1 (28.4% vs 65%) in the dNP tissues (from degenerative disc disease subjects) than in the nNP tissues (from scoliosis subjects) (Fig. 2c), indicating the augmented myofibroblast sprouting in dNP. In line with our single-cell transcriptome analysis, cells from either dNP or nNP tissues were found to contain a lower amount of αSMA^+^ cells than FAPα^+^ or FSP1^+^ cells, which was further supported by fluorescence-activated cell sorter (FACS) analysis of isolated NPC (Fig. 2d). FACS also demonstrated an increased myofibroblast population in the dNP compared to the nNP. Notably, nNP [(78.0 ± 11.7)/%] contained a considerable amount of cells expressing intracellular COL1 compared to dNP [(93.4 ± 5.5)/%] (Fig. S4). Under monolayer expansion, dNP cells were more flattened and expressed higher levels of myofibroblastic markers including *ACTA2*, *FAP* and *COL1A1* (Fig. S5). These findings support that human IDD associates with an accumulation of NPC which have myofibroblastic characteristics.

### Enrichment of fibroblastic NPC with hematopoietic features in IDD

**Fig. 3:**
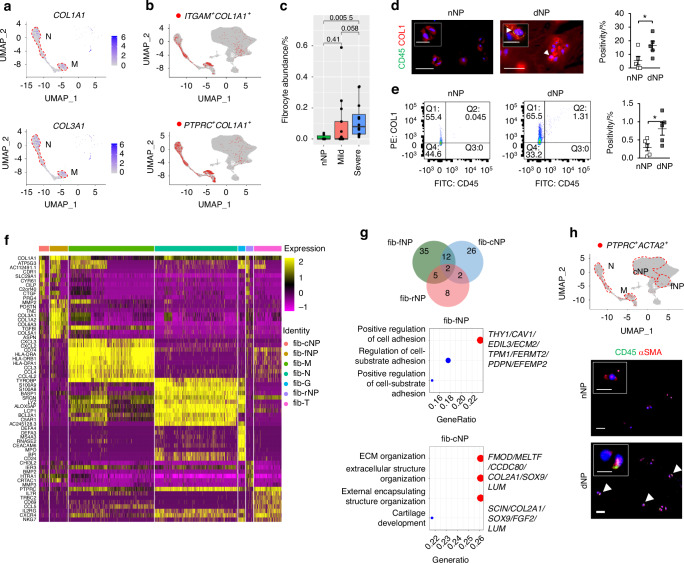
Identification of disc fibrocytes in human disc degeneration. **a** Featured gene plots of fibroblast markers of *COL1A1* (encoding alpha-1 collagen I) and *COL3A1* (encoding alpha-1 collagen III) in disc immunocytes. N: neutrophil; M: Macrophage. **b** Feature plots of expression distribution for monocyte lineage marker *ITGAM* (encoding CD11b) and pan-hematopoietic marker *PTPRC* (encoding CD45) with *COL1A1*. **c** Fibrocyte abundance in disc degeneration. **d** Co-immunofluorescence of CD45 and collagen I (COL1) in non-degenerative human NP tissues (nNP) from scoliosis subject and degenerative NP tissues (dNP) from degenerative disc disease subjects, and quantification of co-expressing cells (*n* = 5). **e** Flow cytometric evaluation of CD45^+^COL1^+^ fibrocytes in isolated human NP cells (NPC). Representative flow cytometry was plotted and the percentages of CD45^+^COL1^+^ cells were labeled (*n* = 5). **f** Heatmap showing and highlighting differentially expressed genes (DEG) for the 7 subtypes of disc degeneration-related fibrocytes. Fibrocytes subsets were classified based on their residing UMAP clusters: ChonNP (fib-cNP), FibroNP (fib-fNP), RegNP (fib-rNP), macrophage (fib-M), neutrophil (fib-N), T cell (fib-T) and granulocyte-like myeloid-derived suppressor cells (fib-G). **g** Venn diagram and GO (gene ontology) analysis of the DEG from NPC-enriched fibrocytes. **h** UMAP distribution of *PTPRC*^+^ (encoding CD45)/*ACTA2*^+^ (encoding αSMA*)* cells and their immunodetection in NP tissues. Arrowhead: CD45^+^αSMA^**+**^ cell; sale bar: 50 μm. Insert: representative staining micrograph showing positive cellular signals; scale bar: 12 μm. *P < 0.05 by two-tailed unpaired *t*-tests

Previous histological studies suggested that immune cells such as CD68^+^ macrophages and neutrophils may infiltrate into the NP during disc degeneration.^19^ In the single cell analysis, immunocyte-like populations could be found at all degeneration grades, expressing markers for neutrophils (*LYZ* and *HLA-DRA*), macrophages (*CD163* and *CD68*), G-MDSC (*ITGAM* and *MPO*) and T cells (*TRAC* and *TRBC2*) (SI file 1). Interestingly, we found that *COL1A1*, *COL3A1* and *POSTN* were also expressed in the immunocytes, predominantly the neutrophil and macrophage populations, albeit at a considerably lower level compared with FibroNP (Figs. 3a and S6). These *COL1A1*^*+*^ immunocytes expressed myeloid lineage genes (e.g., *S100A9*, *PTPRC, ITGAM* and *ITGB1*), *S100A4*, and ECM genes *VIM*, *COL1A1* and *FNDC3A*, but not macrophage marker *CD115* or T-cell marker *CD90* (*THY1*) (SI file 2), consistent with the classification of fibrocytes.^20^ Fibrocytes are conventionally defined as a rare subpopulation of monocytic progenitors that co-expresses collagen I and III in addition to hematopoietic markers CD11b, CD45 or CD34.^21–23^ While we could identify cells showing a co-expression of CD34 with COL1 or αSMA in the dNP tissue, the integrative scRNA-seq analysis indicated the *CD34*^+^*COL1A1*^+^ or *CD34*^+^*ACTA2*^+^ cells were in fact mostly enriched in the endothelial cell and pericyte clusters but not the immunocyte cluster (Fig. S7). We further identified *COL1A1*^+^ subpopulations co-expressing *ITGAM* (coding CD11b) (1 094 cells) or *PTPRC* (coding CD45) (1738 cells) (Fig. 3b). The abundance of these subpopulations increased with degeneration severity and occupied up to 0.1% cell population in the degenerative group (Fig. 3c). Immunostaining and flow cytometry analysis demonstrated the presence of CD45-expressing cells in the degenerative samples, all of which co-express COL1. These CD45^+^COL1^+^ cells were increased in the dNP tissue (16.4% vs 5.4%, Fig. 3d) or dNP-derived cell culture (0.80% vs 0.30%, Fig. 3e) compared to the nNP controls and adopted an elongated morphology (insert, Fig. 3d). The combined single cell analysis and in vitro validation therefore support the presence of fibrocytes in degenerative IVD.

Mapping of the *PTPRC*^*+*^*COL1A1*^*+*^ and *ITGAM*^*+*^*COL1A1*^*+*^ cells indicated the enrichment of fibrocytes within the NPC clusters, present mostly in the FibroNP (7.5%, as fib-fNP), ChonNP (4.2%, as fib-cNP) and RegNP (3.0%, as fib-rNP), in addition to the immunocyte clusters of macrophages (35.4%, as fib-M), neutrophils (34.2%, as fib-N), T cells (11.4%, as fib-T) and G-MDSC (3.0%, fib-G) (Table S2). By comparing these fibrocyte populations, we identified specific gene markers (SI file 3) and examined their expression (Fig. 3f). *GNB1* and gluconeogenesis-related gene *ALDOA*, together with marker genes of *COL1A1* and *ITGAM*, were enriched in both immunocytes- (fib-M/N) and NPC-clustered (fib-cNP/fNP) fibrocytes. Within immunocyte-clustered fibrocyte subpopulations (fib-M/N/T/G), a total of 47 genes (e.g., *PTPRC* and *CAPZA1*) were considerably enhanced and mainly linked to the actin organization and immune process (Table 2 and Fig. S8). *RORA* was dominantly expressed in fib-T. Fib-M-related genes *LGALS1* and *LGALS3BP* have been linked to monocyte-derived fibrocyte differentiation.^24^ 43 genes (e.g., *COL3A1*, *ASPN* and *ITGB5*) that are commonly expressed in the three NPC-derived fibrocyte subsets are related to the response to TGF-β (Fig. S8). This finding is consistent with the notion that, similar to other fibrotic diseases, disc fibrocyte maturation may involve TGF-β-mediated endothelial- mesenchymal transition.^25^ We also performed GO analysis for DEGs specific to the NPC-fibrocytes (Table 3). 35 genes distinctly enriched in fib-fNP were mostly related to cell adhesion function. 26 genes enriched in the Fib-cNP were related to matrix organization. Two degeneration-featured genes, *HTRA1*^26^ and *ANGPTL4*^27^ were distinctly expressed in the NPC-fibrocytes (Fig. 3g). Fibroinflammatory molecules TGFBI and SPP1 from the disc fibrocytes were inferred to act on the FibroNP and stimulate the fibrotic process in CellChat analysis (Fig. S9). Fibrocyte-FibroNP communication may also rely on POSTN-ITGB5. Moreover, *ACTA2* was found expressed in *PTPRC*^+^ FibroNP cells (Fig. 3h), supporting their potential in myofibroblastic differentiation.^24,25^ Consistent with the scRNA-seq finding, co-immunostaining showed a markedly higher expression of CD45 and αSMA in the dNP compared to the nNP (Fig. 3h). We also observed αSMA^+^ cells negative for CD45.

**Table 2:** Analysis of DEGs for immunocytes-clustered fibrocytes

DEGs	Immunocytes-clustered fibrocytes			
	fib-M	fib-T	fib-G	fib-N
Featured genes	*CSTB LGMN ANXA2 DAB2 LGALS1* *CTSL PLD3 CST3 NRP2 LMNA* *MGST3 PDE4DIP*	*RORA*	*NUCB2* *DUT*	N/A
Commonly shared positive genes	*LAPTM5 PTPRC CXCR4 CFLAR HCLS1 PLEKHA2 FAM49B CELF2 SRGN ARHGDIB LCP1 EVI2B FMNL1 COL1A1 GMFG ITGB2 TMSB4X SAMSN1 MT-ND1 RAC2 B2M MT-ND3 SLA WDR26 FAM107B DEDD2 PFN1 MT-ND4 CFL1 FYB1 HLA-B LASP1 WIPF1 PPP1R18 GNB1 HLA-E HLA-A MSN MT-ATP8 MYH9 RAB8B ATP5E BAZ1A ARPC2 CAPZA1 MT-ND6 PABPC1*			
Commonly shared negative genes	*GLT8D2 NCKAP1 NGEF FAP TMEM98 SOX5 DIXDC1 CD320 GSTM3 PDGFRL SERPINA1 SERTAD4-AS1 KDELR3 BARX1 TGM2 ZNF385D CFB FKBP7 ECM2 GPC6 CAV2 CHPF CDH19 AOPEP PERP LARP6 CNN3 CCDC85B TUBB2B MIR100HG MPP6 MAGI2-AS3 TIMP4 GFPT2 ARL3 MDFI SCIN SMOC1 VASN DPT LAMB2 CHST3 PPFIBP1 ACKR3 HSPA2 EVA1B GOLM1 METRN GNG11 WWTR1 SGCB ITGA10 MELTF PYURF THY1 FXYD1 LY6E EFEMP2 SNAI2 SSPN VGLL4 ADPRHL1 FHL2 DDR2 LTBP3 CAVIN3 RGS3 CILP2 ANGPTL2 PAX1 FSTL1 FGFRL1 P4HA2 FAM114A1 FAM3C MRC2 ANGPTL5 TMEM45A COX7A1 ANGPTL4 MXRA7 C1R H19 DANCR COL6A3 RHOBTB3 BAMBI TPM1 NFIA TRPS1 FKBP10 MXRA8 COL5A2 MEG3 CRIP2 TCEAL2 RCN3 COPZ2 IFITM3 CDO1 PDPN ID1 NRN1 TCF4 ID4 PDLIM4 SIVA1 ERRFI1 ADIRF ENPP1 PPIC EDIL3 FOXC1 TPM2 FGF2 NENF FERMT2 NOVA1 PCOLCE CHAD APOD ATP5IF1 RCN2 SLC39A14 EMP2 TMEM59 MAGED2 GPRC5A SBDS COL11A2 PRDX2 WDR83OS ISLR CALD1 AK1 CAVIN1 FHL1 COL6A1 BNIP3 TM4SF1 FXYD6 C1S CLEC3A FBXO2 SOX9 TIMP3 RRBP1 CAV1 MYDGF SERPINA5 UAP1 PPDPF TNFRSF11B SPARC ITM2A FN1 CP EID1 LINC00632 RBP4 BEX3 MT1M KRT10 SERPING1 CPE VCAN VKORC1 PLOD2 PDIA6 CAPS COL6A2 SNHG5 KCNMA1 PMEPA1 MFGE8 COL9A3 SOD3 RABAC1 TNFRSF12A CCDC80 C12orf57 CCN1 DST HAPLN1 PRDX4 COL11A1 GPX3 PLAC9 COL2A1 PPIB FMOD HTRA1 CLU S100B NUPR1 TSC22D1 ZFAS1 ABI3BP SERPINA3 PCOLCE2 OGN S100A13 SERPINE2 PRELP IGFBP7 DSTN SSR4 AEBP1 C11orf96 MIF MIA CNMD SELENOM NNMT S100A1 BGN CCN2 SCRG1 MT1G RPS26 FGFBP2 SNORC ACAN ECRG4 LUM CRYAB DCN TIMP1 MGP NDUFA4L2 MT2A MT1X RPLP0 MT1E COMP RPL7*			

*PTPRC*^+^*COL1A1*^+^ or *ITGAM*^+^*COL1A1*^+^ fibrocytes are subdivided into two major clusters based on their UMAP distribution: immunocytes-clustered fibrocytes and NP cells (NPC)-clustered fibrocytes. Immunocytes-clustered fibrocytes contains fib-M (Macrophage), fib-N (Neutrophil), fib-T (T cell) and fib-G (Granulocyte-like myeloid-derived suppressor cells, G-MDSC). Differentially expressed genes (DEGs) of the different fibrocytes subsets are shown

**Table 3:** Analysis of DEGs for NPC-clustered fibrocytes

DEGs	NPC-clustered fibrocytes		
	fib-cNP	fib-fNP	fib-rNP
Featured genes	*PLOD2 FGFBP2 SPARC FMOD* *HTRA1 TIMP3 ANGPTL2 DPT* *MELTF CCDC80 PCOLCE2* *FHL2 OGN CILP2 GFPT2 SCIN* *PRELP SERPINA5 CPE* *HAPLN1 SCRG1 COL2A1* *ANGPTL4 ABI3BP SOX9 COL5A2 FXYD6 P4HA2 SMOC1 FGF2* *NRN1 MXRA7 CDO1 FSTL1 PPIC* *SERPINA1 ID4 TUBB2B TMEM45A* *LUM CDH19 FAM114A1*	*COL6A3 COL5A2 COL6A1 DPT FSTL1* *SPARC THY1 FAP COL6A2 ISLR CALD1 ANGPTL2 HTRA1 PCOLCE MXRA8* *C1R CAV1 EDIL3 CAVIN1 CNN3 ECM2* *TPM1 PPIC FKBP10 DDR2 PLOD2* *GOLM1 SMOC1 SSPN FAM114A1* *PDGFRL FN1 ERRFI1 TIMP3 FERMT2* *PDPN GLT8D2 MXRA7 SLC39A14 BGN NCKAP1 CAVIN3 ANGPTL4 DST* *WWTR1 SGCB DIXDC1 P4HA2 C1S* *TCF4 RCN3 ACKR3 EFEMP2 IGFBP7*	*DCN FAP NNMT FN1 MFGE8 GFPT2 CLU SERPINE2 SLC39A14 ANGPTL4 GPX3 ERRFI1 SOD3 C11orf96 CDO1 ACKR3 HTRA1*
Commonly shared positive genes	*COL1A1 COL3A1 COL1A2 ASPN TGFBI CTSB CTGF MT-ATP8 ATP5E ALDOA CDR1 HTRA1 ITGB5 MATR3 AC112491.1 ATP5G2 CYR61 ATP5J ATP5H HMOX1 NBEAL1 ATP5G3 ITGAM ATP5A1 ATP5F1 ATP5B ANKH LRRC75A ZNF90 TRAM1 ATP5L CERS2 DUSP5 PPP3CA PXDC1 CDK2AP1 OAT CKS2 ANGPTL4 SLC1A4 LDHA ZFAND5 MAN1A2*		
Commonly shared negative genes	*RPL21 MALAT1*		

NPC-clustered fibrocytes contains fib-fNP (FibroNP), fib-cNP (ChonNP), fib-rNP (RegNP). Featured DEGs (differentially expressed genes) of each subset of NPC-clustered fibrocytes that were negatively expressed in the immunocytes-clustered fibrocytes are shown

**Fig. 4:**
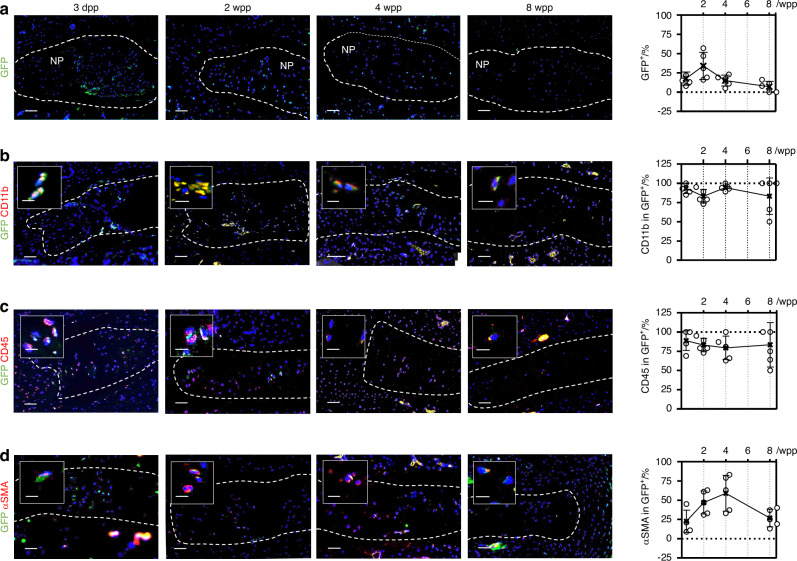
Hematopoietic fibrocytes contribute to myofibroblastic NP cells. Tail disc puncture was performed on CD11b-DTR mice, in which monocytes express green fluorescent protein (GFP) under the CD11b promoter, to induce progressive disc degeneration and fibrosis. **a** Immunostaining and quantification of GFP expression in the degenerative caudal discs of CD11b-DTR in 3 days (dpp) to 8 weeks post-puncture (wpp) window. Co-immunostaining of monocytic marker CD11b (**b**), hematopoietic marker CD45 (**c**) and myofibroblast marker α-smooth muscle actin (αSMA) (**d**) with GFP and quantification of marker positivity (%) in the GFP^+^ cells. Counting was conducted in five representative sections, and expressed as the mean ± S.D. Sale bar: 50 μm. *P < 0.05, One-way ANOVA with Bonferroni post-hoc test

**Fig. 5:**
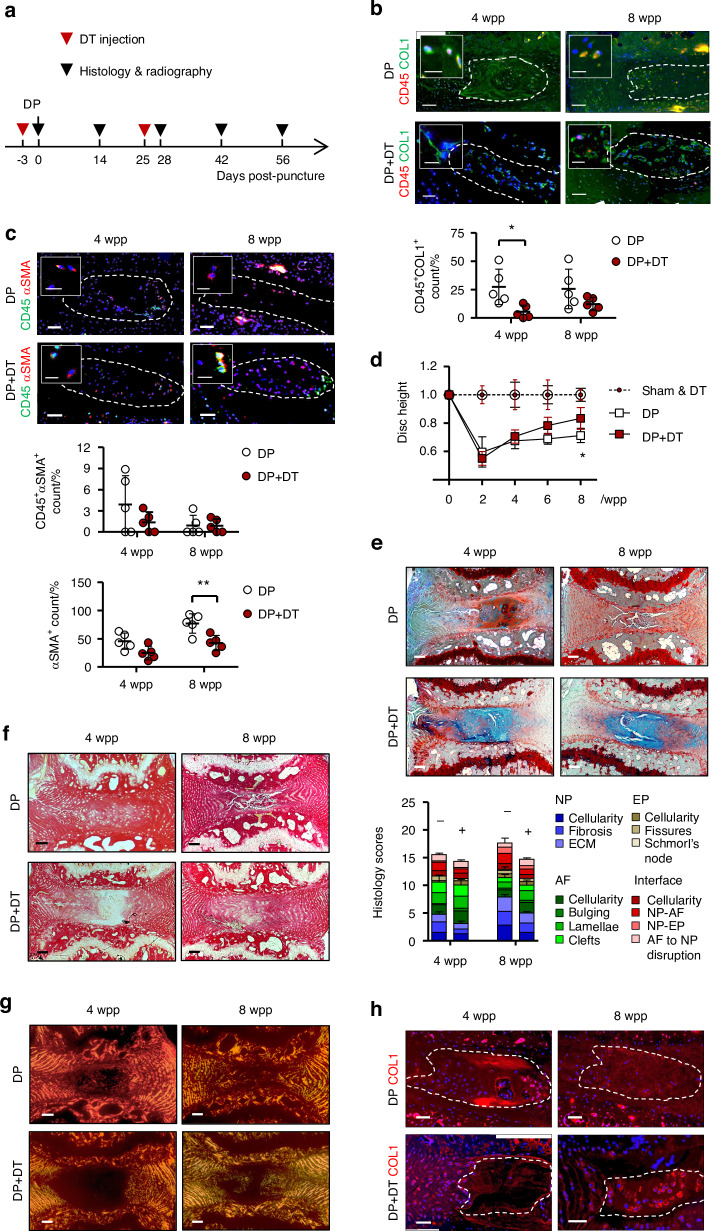
Fibrocyte ablation suppresses disc degeneration and NP fibrosis. **a** Time-line illustration for diphtheria toxin (DT) administration and disc puncture surgery on CD11b-DTR mice (*n* = 5). PBS serves as vehicle control. **b** Co-staining of CD45 (red) with collagen I (COL1, green) and (**c**) CD45 (green) with α-smooth muscle actin (αSMA, red), and the dual-positive NP cells (NPC) counts (*n* = 5). **d** Time course assessment of disc height changes (*n* = 5 per group). **e** FAST staining and degeneration scoring based on compartmental deformation in NP, annulus fibrosus (AF), endplate (EP) and interface. **f** Picrosirius red staining and (**g**) polarized microscopy. **h** COL1 immunostaining. In immunofluorescence, nuclei were counterstained by DAPI. Scale bar: 50 μm, insert scale bar: 12 μm. DP disc puncture, wpp weeks post-puncture. NP regions are encircled (dashed lines). Data are expressed as mean ± S.D from five independent experiments. Two-way ANOVA with Bonferroni post-hoc test, *P < 0.05

Given the fibrocytes being a lineage of monocytes expressing CD11b, we studied their temporospatial distribution in puncture injury-induced disc degeneration in CD11b-DTR mice, in which the expression of EGFP-diphtheria toxin (DT) receptor fusion protein is controlled by the *ITGAM* promoter, thereby directing transgene expression and expressing membrane-localized green fluorescence protein (GFP) in monocytes/macrophages.^23,28^ Normal mouse NP, which contains predominantly notochordal cells,^4^ was negative for GFP expression (Fig. S10). Annulus puncture injury led to disc degeneration with NP fibrosis.^3,4^ We observed an emergence of GFP^+^ cells in the NP (~15% of NPC population) by 3 days after puncture, which continued to peak at roughly 35% by 2 weeks post-puncture (wpp) and gradually subsided thereafter (Fig. 4a). GFP^+^ cells were also observed in the annulus fibrosus (AF) (Fig. S10c). We could observe GFP^+^ cells that barely expressed CD11b in the NP at 2wpp (Fig. 4b), possibly because of a longer turnover of GFP compared to CD11b.^28^ Up to 95% of the GFP^+^ NPC were CD45-positive, indicating their myeloid origin (Fig. 4c). In line with previous report of fibrocyte differentiation into myofibroblast, we observed αSMA expression in nearly 60% GFP^+^ cells at 4 wpp (Fig. 4d). The abundance of these dual-positive cells decreased significantly by 8 wpp, indicating that the emergence of fibrocytes might be transient or they underwent differentiation. Furthermore, we detected CD45^+^COL1^+^ and CD45^+^αSMA^+^ cells in the punctured disc (Fig. 5b, c), and the amount of CD45^+^αSMA^+^ NPC showed a two-fold reduction by 8 wpp compared to 4 wpp. These findings collectively support the notion of fibrocyte infiltration into the degenerating discs and adopting a myofibroblastic phenotype (CD45^+^αSMA^+^) during NP fibrosis.

### Fibrocyte depletion alleviates puncture-induced NP fibrosis

We tested whether depletion of CD11b-expressing cells by diphtheria toxin (DT) administration^28^ could lead to a reduction of fibrocytes in the puncture induced IDD. We first examined the frequency and dosage of DT injection to enable extended depletion while minimizing lethality due to compromised immunity (Table S3). The number of CD11b^+^ monocytes were largely reduced in peripheral blood (>50%) after twice administration of DT at 10 μg/kg (Fig. S11). For the IDD model, the first injection was performed 3 days prior to the puncture surgery (Fig. 5a). We found a marked decrease of the GFP^+^ cells in the NP at 2 and 8 wpp, supporting the effect of DT on the transgene expressing cells (Fig. S11). Interestingly, a lower number of CD45^+^COL1^+^ cells was observed in the NP after the second dose of DT injection by 4 wpp when compared to vehicle control (Fig. 5b). The amount of CD45^+^αSMA^+^ NPC also tended to decrease although the difference is not statistically significant (Fig. 5c). These findings indicate that DT administration caused a depletion of monocytic fibrocytes in the NP which emerged during injury-induced IDD.

We questioned if the loss of the myeloid-derived fibrocytes leads to a modification of IDD and NP fibrosis. Puncture induced IDD showed a significant disc height loss by 40.4% at 2 wpp and 28.4% at 8 wpp (Figs. 5d and S12). DT administration could induce a gradual recovery and reduce the disc height loss to only 17.2% at 8 wpp. Disc integrity assessed by FAST staining^3^ showed a prominent improvement with DT administration at both 4 and 8 wpp, including reduced lacune formation, preserved NP-AF boundary, and increased alcian blue intensity in the NP (Fig. 5e). Moreover, alcian blue staining in the NP of the DT-injected mice was largely maintained, implying that the loss of proteoglycans was prevented. The amount of myofibroblastic NPC positive for αSMA steadily increased after puncture and took up to 76% of the whole population by 8 wpp (Fig. 5c). DT administration could lower its positivity to 41%. It is noteworthy that the majority of αSMA^+^ cells detected in the punctured discs, in particular in the DT-treated group, was negative for CD45 (>90%). Picrosirius red staining and immunostaining of COL1 further consolidated a decrease in fibrillar collagens (Fig. 5f–h). Pericellular expression of COL1 was noted in the NP after DT administration. Collectively, these findings demonstrated that IDD progression is dependent on a function of myeloid-derived cells where the CD45^+^COL1^+^ fibrocytes and αSMA^+^ myofibroblastic cells may mediate NP fibrosis.

## Discussion

NPC are conventionally defined as collagen II-, aggrecan- and Sox9-expressing cells. Recent studies of single-cell transcriptome have indicated cell heterogeneity in the human NP, which contains cell populations expressing notochordal, chondrogenic, and fibroblastic phenotypes.^7,11–14^ Our meta-analysis of the human disc scRNA-seq datasets has outlined the commonly featured NPC hierarchy and stratified their fibroblastic subtypes. *COL1A1*^+^ FibroNP is previously defined as adhesion/fibroNPC^12^ or C4 NPC.^7^ The FibroNP population was not reported in Wang et al scRNA-seq dataset, possibly because their cells were derived from the whole disc tissue without NP isolation and therefore may have dominated by *COL1A1*^*+*^ AF cells.^11^ Nonetheless, they defined the FibroNP genes (*POSTN*, *COL1A1*, *COL3A1* and *TMSB4X*) in MK167^+^ progenitor and NP progenitor cells, implying the fibroblast presence within the two NP subtypes. Han et al found a small fraction of fibrochondrocyte progenitors in the NP, which partially constitute the aforementioned two NP subsets. The regulatory NPC and C1/3 chonNPC subtypes reported by Tu^12^ and Han^7^ are merged to the RegNP cluster in this study, sharing the expression of *MMP3*, *CHI3L2*/*1* and *GPX3*. The CyclingNP cluster is a pool of *TOP*2A^+^ cells fraction from the *COL2A1*^*+*^ ChonNP or *COL1A1*^*+*^ FibroNP subsets, implying they may be derived from a common replicative progenitor origin. Pseudotime ordering of the NP clusters and velocity analysis may help construct their relationship.

The combination of collagen production, in particular collagen I, and the expression of leukocytic common antigen (CD45) or one of the hematopoietic or myeloid antigens (e.g., CD34 or CD11b) is considered as a sufficient criterion for fibrocyte identity.^22^ Our data show that *CD34*^*+*^*COL1A1*^+^ cells are mainly enriched in the endothelial cells and pericytes subpopulations. *ITGAM* (CD11b) or *PTPRC* (CD45) is therefore preferable to CD34 as the marker of fibrocytes in human NP tissues. Notably, the *PTPRC*^*+*^*COL1A1*^*+*^ and *ITGAM*^*+*^*COL1A1*^*+*^ cells express many common genes, and that *PTPRC*^*+*^ cells are more abundant and have a wider UMAP distribution than *ITGAM*^*+*^ cells. This is consistent with the suggestion that pan-hematopoietic marker CD45 is a more representative fibrocyte marker compared to CD11b.^22^ Our data indicate that the fibrocytes constitute 10%–20% of the NPC in the degenerative samples, in agreement with the low abundance of circulating fibrocytes (CD45^+^COL1^+^) found in lung (1.3 ± 1.6 cells/mm^2^) and kidney fibrosis (80 cells/mm^2^ or 12% positivity).^20,29^ Pronase digestion was commonly deployed for releasing single cells from the NP in scRNA-seq and flow cytometry assays.^30^ However, the digestion can also result in extensive membrane protein degradation, and thereby affect the antibody recognition of surface molecules.^31^ This may in part explain the discrepancy in measurements of CD45^+^COL1^+^ cells between in situ immunohistochemistry and flow cytometry.

In addition to the NP clusters, the *ITGAM* ^*+*^*COL1A1*^+^ or *PTPRC*^*+*^*COL1A1*^+^ cells are mostly located within the macrophages, neutrophils and T cells clusters, implying the fibrocytes may be derived from multiple sources or differentiation routes of the immune system. A potential causal relationship between blood immune cells and IDD have been suggested.^32^ Interestingly, differentiation of CD11b^+^CD115^+^Gr1^+^ monocytes into fibrocytes could be dependent on CD4^+^ T cells.^33^ Immune cells can infiltrate through focal defects in cartilaginous endplate (EP) and AF and advance the fibroinflammatory process.^34^ The granulocyte-like myeloid-derived suppressor cells (G-MDSCs), shown in cardiac fibrosis with myofibroblast activation capabilities, may contribute to T-cell suppression and ROS production in IDD.^12^ Monocyte-derived inflammatory macrophages could differentiate into myofibroblasts^35,36^ and were identified in the NP of degenerative discs.^19,37^ Interestingly, CD11b^+^CD45^+^ monocytes were previously reported to support the proliferation and colony forming ability of cultured MSCs.^38^ Whether the monocytic cells infiltrated into the degenerating discs may exert a similar effect on local mesenchymal progenitors to promote fibrogenesis or repair is not clear. Moreover, CCR2^+^ monocytes have been identified in the degenerative IVDs from herniation patients and constitute the majority of CD11b^+^ and F4/80^+^ cells.^39^ However, as reported in myocardial infarction model,^40^ loss of CCR2 expression is required for fibrocyte differentiation of the infiltrated monocytes. This is in line with our observation that the disc fibrocytes are negative for *CCR2*. In a mouse model of renal fibrosis where CCR2 is depleted, migration of circulating fibrocytes to the kidney was interfered.^28^ Whether CCR2 plays a role in monocyte infiltration and hence differentiation into disc fibrocytes await to be determined.

Fibrocytes could be derived not only from local monocyte differentiation but also migrated from peripheral blood in the form of partially differentiated collagen-producing cells.^22,28^ Recruitment of monocytes and peripheral blood fibrocytes may rely on disc neo-vascularization. Normally, blood vessels recede from the outer AF and the EP in adults. However, under degenerative conditions the NP can become re-vascularized and blood vessel infiltration is frequently observed and spatially associated with macrophage infiltration.^41^ Most of the dNP tissues in this study for scRNA-seq analysis were dissected from extruded NP of herniated discs that normally show pronounced neo-vascularization.^42^ The increased number of GFP^+^ cells found in the AF and EP of the punctured CD11b-DTR discs may indicate the infiltration through the AF and EP routes. CD34^+^αSMA^*+*^ cells, presumably marking the endothelial cells and pericytes, could in fact be detected in the human dNP, supporting the presence of neo-vascularization. Studying disc vascularization and its relation to the emergence of disc fibrocytes may provide further insights into the pathway of NP fibrosis.

Trafficking fibrocytes express chemokine receptors CCR5,^43^ CCR7^22^ and CXCR4.^22,44^ Studies have reported increased CCR5 expression in AF and NP cells^45^ and chemokines CCL2/7 in dNP.^46^ Bone marrow cells, such as CD146^+^ MSCs,^47^ may have a potential to migrate into the NP, possibly by control of SDF1.^48^ CCL5 could interact with CCR1/3/5 in NPC and was up-regulated under inflammatory stimuli.^49^ CXCR4 is a receptor of SDF1^50^ and CXCL12,^44^ which was found upregulated in degenerated discs and implicated in disc angiogenesis^50^ and cellular apoptosis^51^ via PI3K/NF-κB pathway. Notably, the combination of CXCR4 and COL1 expression can identify fibrocytes in idiopathic pulmonary fibrosis.^29^ As the disc fibrocytes highly express *CXCR4* and *CCR1* (Table 2), they might therefore communicate with the ProgNP population via the CXCL12-CXCR4 axis. Future study may test if CXCL12 has a function to recruit disc fibrocytes.

Tissue fibrosis is mediated by myofibroblast from local or extrinsic sources^8^ and fibrocytes may serve as one of the progenitors of myofibroblasts.^22,25^ Expression of αSMA is the hallmark of mature myofibroblasts and essential to their contractility.^18^ Expression of αSMA in *COL1A1*-expressing FiboNP cells could therefore indicate their myofibroblastic property.^25^ In addition to myofibroblasts, expression of *ACTA2*/αSMA, in combination with *TAGLN* (encoding transgelin) and *MCAM* (encoding CD146), can also mark pericytes. We find that the fibrocytes are not associated with the pericyte cluster, therefore the expression of αSMA found in the CD45^+^ and CD11b^+^ cells in the dNP is more likely related to myofibroblastic conversion rather than detection of pericytes. However, infiltrating pericytes can in fact contribute to scar-forming myofibroblasts in kidney and subretinal fibrosis.^8,52^ Lineage tracing model can be used to delineate the contribution of pericytes to myofibroblasts in NP fibrosis in future.

FAPα is a well-known biomarker of (myo-)fibroblasts, and together with FSP1 it can label most of the COL1-producing cells in the bone.^53^ FSP1 marks an active fibroblast population and represents a non-overlapping fibroblast entity to αSMA^+^ subtype in heart, kidney and skin fibrosis disease.^54^ Our scRNA-seq analysis show that *FAP*^*+*^ or *S100A4*^*+*^ cells are not only found in the FibroNP but also in the ChonNP, although their overall abundance is not associated with degeneration severity (Fig. S13). We speculate that the ChonNP cells might be primed to become the FibroNP cells in the degeneration process. This notion is consistent with the finding of NPC transformation into a chondrogenic and later fibroblastic phenotype in a previous notochordal cell tracing model study.^4^

In this study, a vast number of αSMA^+^ cells in both human degenerative IVDs and mouse punctured discs are not positive for CD45 and CD11b. Also in the CD11b-DTR mice where the monocytes and their derived lineages (including fibrocytes) were depleted,^28^ αSMA expressing cells could still be observed (>55%, 2 wpp in Fig. 5c) in the degenerative NP. This may be due to the existence of non-hematopoietic origins of the myofibroblast pool, such as those derived from resident NPC.^4^ Alternatively, fibrocytes may lose CD45 and CD11b expression during fibrocyte differentiation.^35^ We also note the presence of residual CD45^+^COL1^+^ cells and GFP^+^ cells after DT administration. This likely arises from the incomplete monocyte ablation and a continuation of fibrocyte recruitment.

Fibrocyte-to-myofibroblast conversion acquires activation of TGF-β/SMAD signaling,^55^ and interleukin-18 receptor 1^56^ and muscarinic receptor M3^57^ are shown to promote the fibrocyte formation and contractile function. Moreover, fibrocytes can secret soluble factors such as periostin^58^ and TIMP1^59^ and semaphorin-7A^60^ to regulate myofibroblast activity and collagen expression in pulmonary and intestinal fibrosis.^21^ Class 3 semaphorins expression are found associated with disc innervation and angiogenesis.^61^ Interestingly, TIMP1^62^ and POSTN^63^ are up-regulated in the degenerated discs and enriched in the FibroNP. Our CellChat data suggest that POSTN may interact with ITGB5 of disc fibrocytes. Whether disc fibrocytes regulate NP fibrosis through a paracrine manner awaits to be addressed. Furthermore, the NPC-enriched disc fibrocytes express *HTRA1* and *ANGPTL4*. HTRA1 is a proteolytic enzyme for degrading the matrix either on its own^64^ or by upregulating ADAMTS5^26^ or matrix metalloproteinases, whereas expression of ANGPTL4 is positively associated with IDD severity.^27^ Notably, our data suggest that ANGPTL4 is a major mediator of the intercellular communication between the disc fibrocytes and the CyclingNP, RegNP and FibroNP subpopulations. Their regulatory role in the fibrocytes and NP fibrosis worths to be investigated.

In conclusion, this study implicates a function of myeloid-derived fibrocytes in NP fibrosis, shedding new insight into the cell fate control of disc immunocytes and their modulatory effect on IDD progression. Understanding the effects and regulation of the fibrocytes may potentiate the development of new diagnostic and therapeutic strategies for modifying IDD.

## Materials and methods

### scRNA-seq data acquisition and downstream analysis

Four single cell RNA sequencing data sets (GSE160756, GSE165722, CNP0002664, PRJCA014236) were downloaded and processed for next-step analysis. 31 individual samples were rearranged according to Pfirrmann grade, where grade I samples were classified as non-degenerative NP, Pfirrmann grade II/III as mildly degenerated NP, and Pfirrmann grade IV/V as severely degenerated NP (Table 1). The degenerated NP samples were mainly obtained from patients with disc herniation (*n* = 23), with the rest from burst fracture subjects (*n* = 2). Seurat package (v4.3.0) (was used for comprehensive single-cell analysis with default settings, except those addressed below. This encompassed processes like quality control, normalization, scaling, dimensional reduction, unsupervised clustering, and the recognition of cluster gene markers. FastMNN (mutual nearest neighbor) algorithm implemented in the SeuraWrappers package was used to remove batch effect across samples, resulting in a correction matrix with 50 principal components (PCs). The top 40 principles were used to run the Uniform Manifold Approximation and Projection (UMAP) and cluster cells at a resolution of 0.4. Marker genes were determined with *P* < 0.05, min.pct = 0.25, pct.1 > pct.2, and log(fold-change) > 0.25 by the FindAllMarkers function. CellChat analysis was conducted to identify potential intercellular interactions, and communication probability values for each interaction were calculated.

### Human samples

All procedures described in the present study were approved by the Institutional Ethics Review Board of The University of Hong Kong. IVD tissues were obtained from patients under informed consent. Non-degenerative and degenerative IVDs were harvested from adolescent idiopathic scoliosis (*n* = 7) and lumbar degenerative disc disease patients (*n* = 9) respectively. The age of the patients ranged from 14 to 72 years, and tissue was evaluated with the Pfirrmann classification system (Table S4).

### Cell isolation and culture

NP tissues were carefully dissected away from the cartilaginous EP and AF, identified by their transparent or pale colloidal appearance. The tissues were then rinsed by saline buffer to remove blood contamination and unwanted debris. In severely degenerated IVDs (grade IV–V), where the NP tissues were fibrotic and not distinguishable from the inner AF, only the most central regions of the NP were harvested. Primary NP cells (NPC) were extracted by sequential enzyme digestion with pronase (0.25%, Roche Diagnostics) and collagenase II (600 U/mL, Worthington Biochemical),^30^ and maintained in high-glucose Dulbecco’s modified Eagle’s medium (DMEM) (Invitrogen) supplemented with 10% fetal bovine serum (FBS, Biosera) and 1% penicillin/streptavidin (P/S, Invitrogen) at 37 °C and 5% CO_2_.

### Flow cytometry

Newly extracted NPC were washed by phosphate buffered saline (PBS) and re-suspended in PBS. Cells were fixed in 4% paraformaldehyde (PFA) and blocked by 5% bovine serum albumin (BSA). Permeabilization with 0.1% triton X-100 (Sigma-Aldrich) was applied where necessary. Cells were then treated with primary and secondary antibodies, and signals from cells labeled with conjugated fluorophores were detected by using BD FACS CantoII Analyzer (BD Biosciences). The antibodies used for different flow cytometry analysis are listed in Table S5. Appropriate IgG control fluorescence compensation was applied to avoid false positive signals. Data were further analyzed by BD FACS Diva software (BD Bioscience).

### Real-time quantitative PCR

Total RNA was prepared using RNeasy mini kit (Invitrogen) and cDNA synthesis was performed using High-capacity RNA-to-cDNA kit (Applied Biosystems). PCR was performed using PowerUp SYBR green master mix (Applied Biosystems), and data are presented as expression levels relative to *GAPDH* using the 2^-ΔΔCt^ method. The primer sequences were listed in Table S6.

### Mouse disc puncture model

Tg (ITGAM-DTR/eGFP)34Lan/J mice which express membrane localized green fluorescent protein (GFP) with diphtheria toxin (DT) receptor under the control of CD11b promoter were used for disc puncture. The animal experiments were approved by The University of Hong Kong Committee on the Use of Live Animals in Teaching and Research (CULATR). CD11b-DTR mice (female, 12 weeks of age, *n* = 5 per group and time points) received needle puncture at levels of C5/6 and C7/8 of tail IVDs as described previously.^4^ At 3 days prior to the surgery and 4 weeks after the surgery, these mice were subjected to intraperitoneal injection of saline or DT (10 μg/kg body weight) (Sigma-Aldrich). At assigned end points, mice were euthanized by intravenous injection of sodium pentobarbital (1.2 g/kg), and intact discs with attached vertebral bones were harvested for histological study. Peripheral blood was harvested at day 0, 3, and 7 after DT injection, and number of GFP^+^ monocytes was measured by fluorescence-activated cell sorting (FACS).

### Disc height measurement

Anterior-to-posterior radiographs of tail discs of CD11b-DTR mice or C57BL/6J were taken using a digital radiography machine (Siemens) with an exposure at 25 kV, for 5 s. The radiographs were obtained biweekly in all groups up to 8 weeks. Disc height index was calculated as previously described^4^ and presented as percent relative to the unoperated level of tail disc C6/7.

### Histology and immunostaining

Samples from human and CD11b-DTR mice tissues were fixed with 4% paraformaldehyde in phosphate buffer for 24 h. Decalcification was performed for mouse IVDs using mouse solution. Paraffin-embedded tissue blocks of human NP and mice IVDs were sectioned at 7 μm. For histological staining, sections were deparaffinized and rehydrated, and subjected to FAST staining (Alcian blue, Safranin O, Fast green and Tartrazine stain) (Sigma-Aldrich) as previously described,^65^ or Masson’s trichrome stain and Picrosirius red stain following standard procedures. Polarized microscopy was combined with picrosirius red staining to observe the collagens network in human NP tissue and mouse IVDs.^66^ According to the histomophological changes, disc degeneration in CD11b-DTR mice was scored with reference to Tam’s scoring system.^65^

For immunofluorescence, antigen retrieval was performed following rehydration by incubating with 0.8% hyaluronidase (Sigma-Aldrich) at 37 °C for 30 min and antigen retrieval buffer (100 mmol/L Tris, 5% EDTA, pH 9.5) at 95 °C for 10 min. The sections were blocked in protein block solution (DAKO) for 60 min before incubating with antibodies. Fluorophore conjugated antibodies were used to enable fluorescence detection. Information for these primary and secondary antibodies were summarized in Table S5. The sections were mounted with VECTASHIELD Mounting Medium with DAPI for nuclei staining. Isotype-matched mouse, rabbit or rat IgG was used as negative controls. Tissue sections were imaged by fluorescence microscope (Nikon Eclipse E600), and fluorescent positive cells in five fields of view (FOV) were manually counted in ImageJ with reference to total number of cells.

### Data analysis

Data are presented as mean ± S.D. of four to six independent experiments, as indicated in respective figure legends. Unpaired t-test (two tailed) was used to determine significant differences. Comparisons among multiple groups were assessed using one-way ANOVA with Bonferroni post-hoc test. A *P* < 0.05 was considered statistically significant.

## Supplementary information

Supplementary Information 1-DEGs of 12 disc cells clusters

Supplementary Information 2-DEGs of COL1A1-expressing immunocytes

Supplementary Information 3-DEGs of fibrocytes subtypes

Supplementary Information

## Data Availability

Genes expression matrices for the cell clusters can be found at 10.6084/m9.figshare.26019766.
