# Supplementary material for: Fibrocyte enrichment and myofibroblastic adaptation causes nucleus pulposus fibrosis and associates with disc degeneration severity
Source: Bone Res. 2025 Jan 20;13:10. doi: 10.1038/s41413-024-00372-2 (PMC11743603; doi:10.1038/s41413-024-00372-2)
Supplement: Supplementary file 4 — Supplementary Information [file 41413_2024_372_MOESM4_ESM.pdf]

Supplementary data

Supplementary Figure S1. Association of NPC clusters with disc degeneration severity. (A) Distribution of the five NP cells (NPC) clusters that were identified from integrative single-cell RNA sequencing (scRNA-seq) analysis in different disc degeneration grades. **(B)** Degeneration-related abundance of the FibroNP subsets. P values were calculated based on unpaired t test.

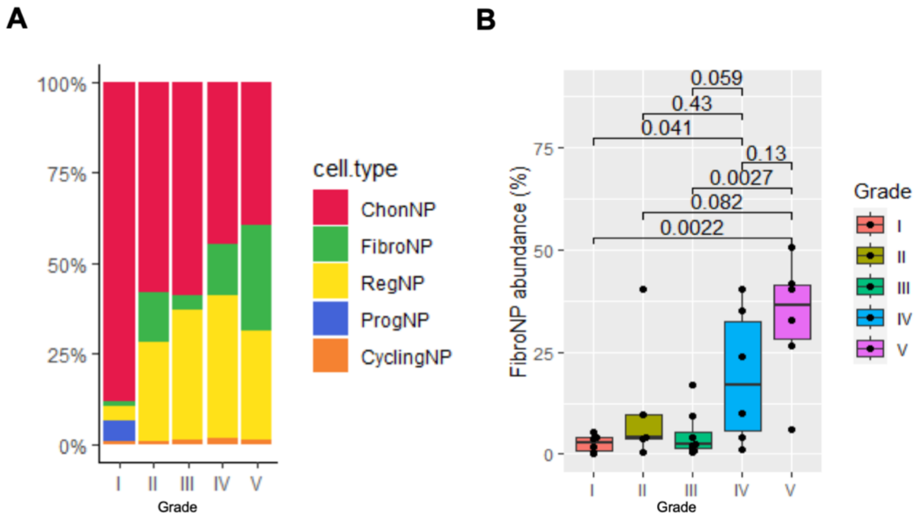

Supplementary Figure S2. Expression of pericyte and endothelial cell markers. UMAP shows the predominant expression of *RGS5* and *CD34* in the pericyte and endothelial cell cluster respectively.

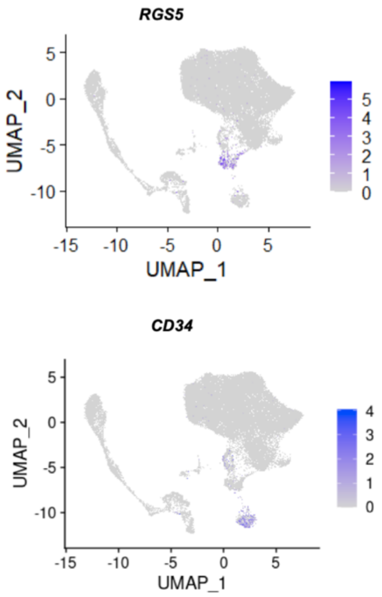

Supplementary Figure S3. Histological evaluation of nucleus pulposus fibrosis in human disc degeneration. Various staining assays were performed on non-degenerative NP (nNP) samples from scoliosis subjects or degenerative NP (dNP) samples from degenerative disc disease subjects: Hematoxylin and eosin staining (H&E); Multichromatic staining of Fast green/Alcian blue/Safranin O/Tartrazine (FAST); Polarized microscopy of Sirius red (PSR); immunofluorescence staining of fibrotic collagen I (COL1, green) and collagen III (COL3, green), and hyaline matrix components of aggrecan (ACAN, green) and collagen II (COL2, red). Arrow head: round-shaped cells; Arrow: spindle-shaped cells. Scale bar: 50μm. Nuclei were counterstained with DAPI (blue).

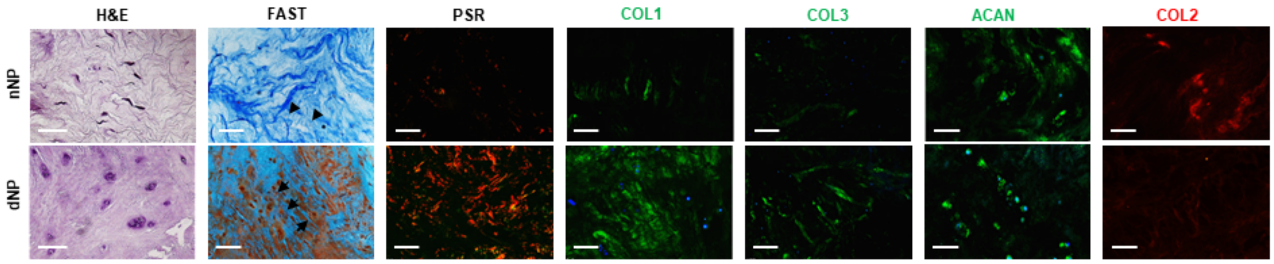

Supplementary Figure S4. Flow cytometry analysis of COL1^{+} NP cells. (A) Representative flow cytometry of isolated human NP cells for collagen I expression (COL1) **(B)** Quantification of COL1^{+} cells (n=3). nNP: non-degenerative NP cells from scoliosis subjects; dNP: degenerative NP cells from degenerative disc disease subjects. Two-tailed unpaired *t*-tests.

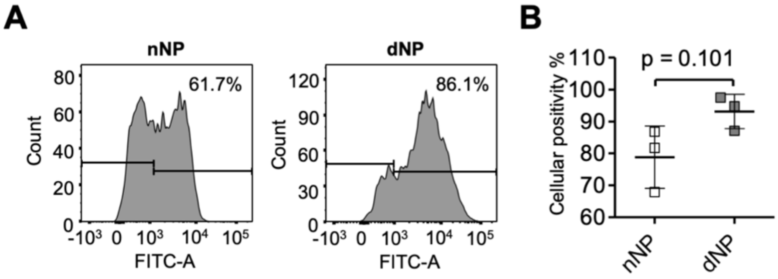

Supplementary Figure S5. Characterization of myofibroblastic phenotype in NP cells culture. (A) Representative immunofluorescence of primary human NP cell culture for (myo-)fibroblast markers. nNP: non-degenerative NP cells from scoliosis subjects; dNP: degenerative NP cells from degenerative disc disease subjects. Scale bar: 50µm. **(B)** RT-qPCR of gene markers of myofibroblast and NP cells, and represented as fold changes in dNP cells over nNP. * $p < 0.05$, ** $p < 0.01$, *** $p < 0.001$ by one sample *t*-tests.

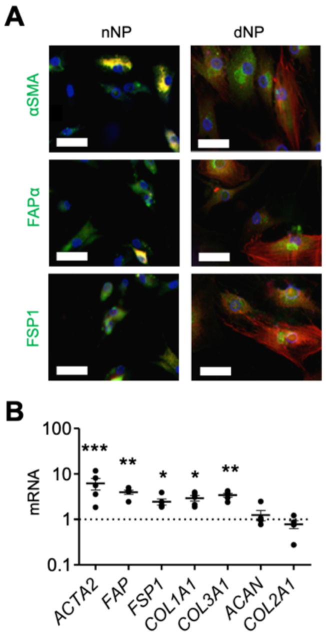

Supplementary Figure S6. Abundance and expression level of *COL1A1* in disc FibroNP and immunocyte populations. FibroNP: *COL1A1* (encoding alpha-1 collagen I chain)-expressing NP cells; G-MDSC: granulocyte-like myeloid-derived suppressor cells.

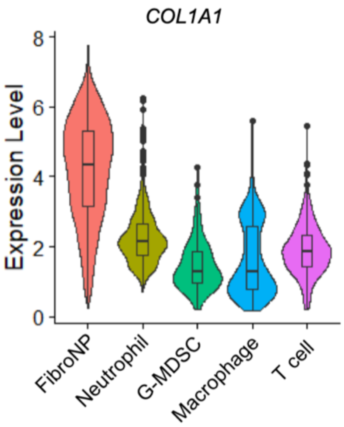

Supplementary Figure S7. Analysis of putative fibrocyte marker CD34. Immunodetection of co-expression for myeloid antigen *CD34* with *COL1A1* (encoding alpha-1 collagen I chain) (**A**) and *ACTA2* (encoding αSMA) (**B**) in NP cells and UMAP distribution in the integrative single-cell RNA-sequencing (scRNA-seq) analysis of four published datasets. nNP: non-degenerative NP from scoliosis subjects; dNP: degenerative NP from degenerative disc disease subjects. CD34 was in green, and COL1/αSMA in red. Scale bar: 50µm. Insert: representative cells micrograph showing positive signals; scale bar: 12µm.

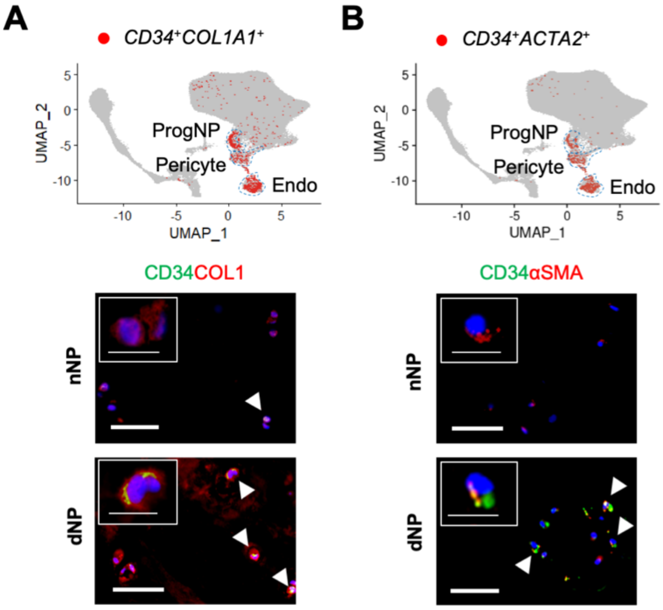

Supplementary Figure S8. GO (gene ontology) functions of immunocyte- and NPC-clustered fibrocytes. Plots of biological processes that were enriched from **(A)** 47 positively expressed differentially expressed genes (DEGs) and **(B)** 236 negatively expressed DEGs in immunocyte-clustered fibrocytes, and **(C)** from 43 positively expressed DEGs of NP cells (NPC)-clustered fibrocytes. Cluster-specific fibrocytes were identified based on their UMAP distribution: macrophage (fib-M), neutrophil (fib-N), T cell (fib-T) and granulocyte-like myeloid derived suppressor cells (fib-G)] and NPC subsets [ChonNP (fib-cNP), FibroNP (fib-fNP) and RegNP (fib-rNP)].

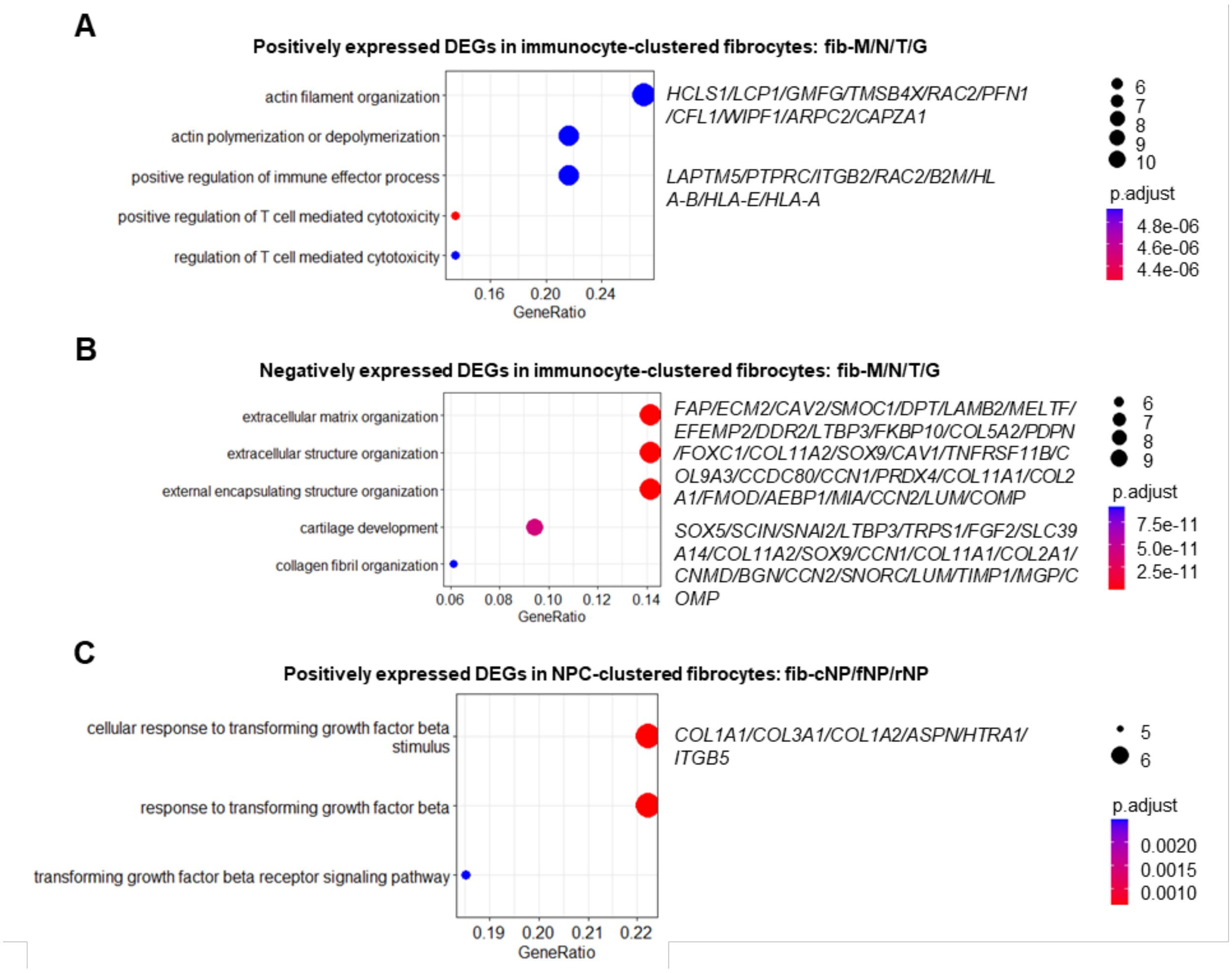

Supplementary Figure S9. CellChat analysis of interaction between disc fibrocytes and the NPC. Bubble plot shows predicted signals sent from (left panel) and received by (right panel) fibrocytes in their interaction with NP cell (NPC) subsets.

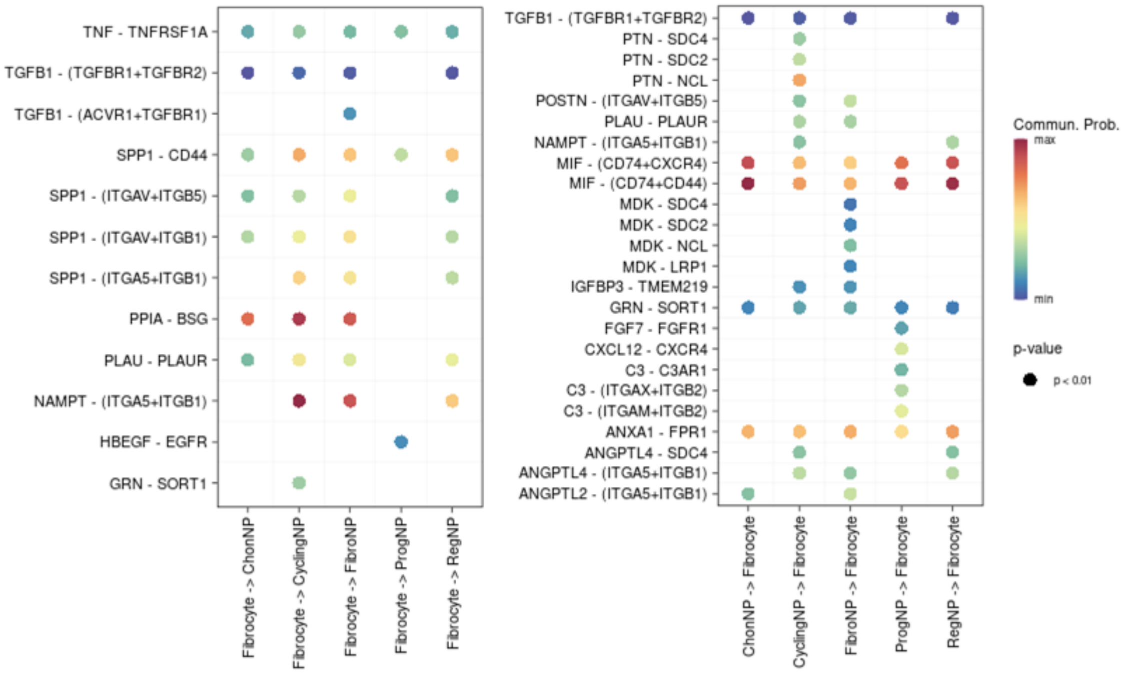

Supplementary Figure S10. Immunodetection of monocytic cells in CD11b-DTR mice. Co-immunofluorescence of hematopoietic marker CD45 and myofibroblast marker α-smooth muscle actin (αSMA) with GFP (expressed by CD11b^{+} monocytes) in **(A)** bone marrow and **(B)** un-operated healthy discs (as sham control in Figure 4). **(C)** GFP expression in annulus fibrosus (AF) of punctured discs. Scale bar: 50µm. dpp: days post-puncture; wpp: weeks post-puncture.

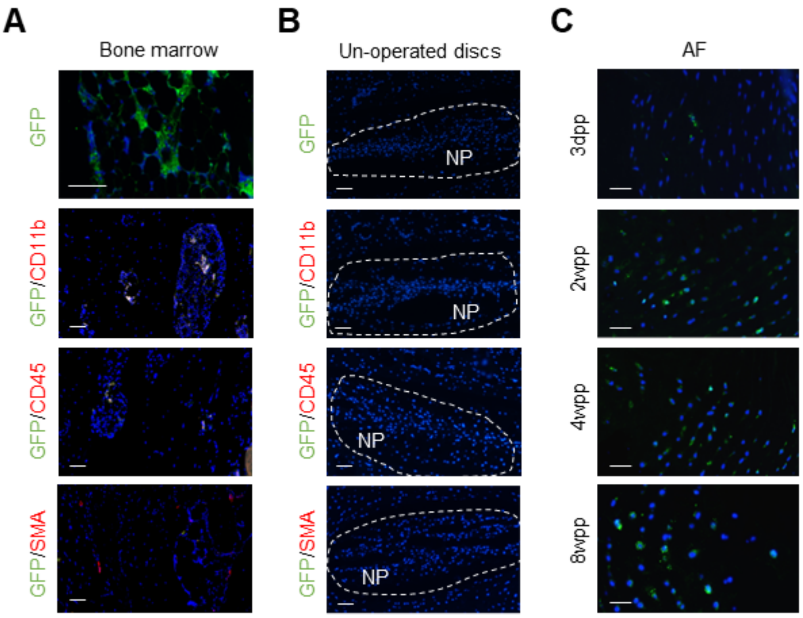

Supplementary Figure S11. Monocyte depletion in CD11b-DTR mice. (A) Representative flow cytometry of $CD11b^{+}$ monocyte in peripheral blood. Diphtheria toxin (DT) was injected at indicated dosages. Isotype control was shown in blue. (B) Representative micrographs for green fluoroscence protein (GFP) staining in punctured discs. DT injection was conducted on CD11b-DTR mice 3 days prior to disc puncture and 4 weeks later at dosage of 10ng/g. Scale bar: 50μm. wpp: weeks post-puncture.

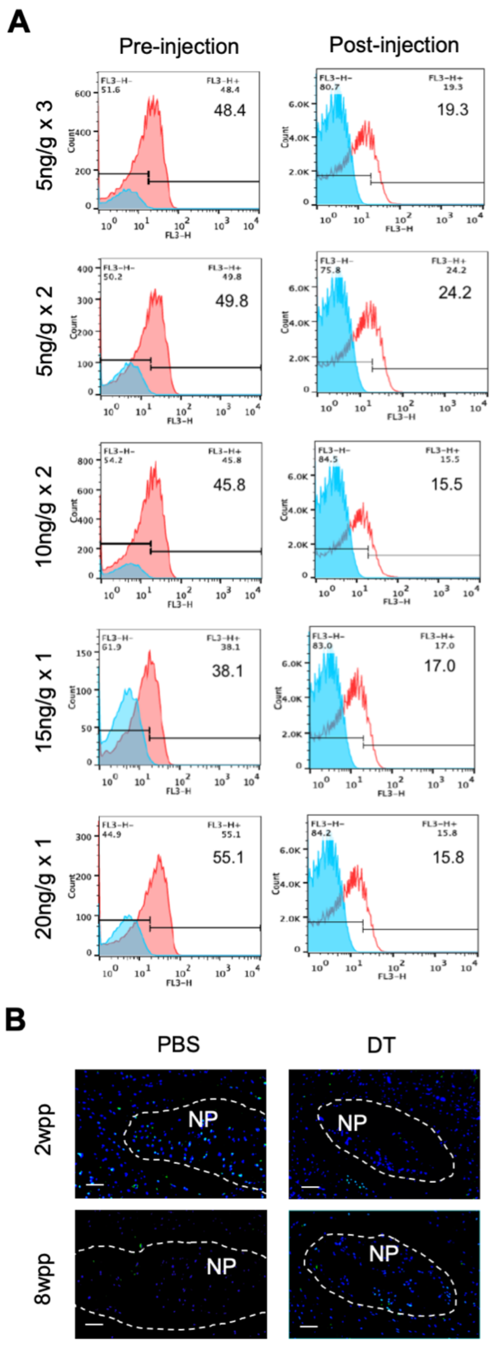

Supplementary Figure S12. Representative disc radiography. The CD11b-DTR mice received disc puncture surgery (DP) at tail coccygeal disc level of C5/6 and C7/8 (n=5 per group). Diphtheria toxin (DT) was administrated via intra-peritoneal injection. PBS serves as vehicle control. The X-ray images were taken for a time-course assessment of disc height changes. Triangle mark: punctured discs.

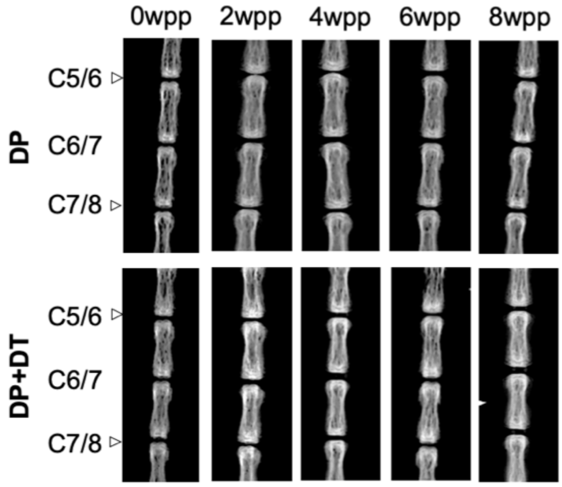

Supplementary Figure S13. Expression of myofibroblastic markers in intergrative single-cell RNA sequencing analysis. (a) Abundance of total *ACTA2, FAP* and *S100A4* expressing cells in human samples of different disc degeneration severity, and **(b)** Abundance of the highly expressing cells (count>3). *P* values were calculated based on unpaired t test. nNP: non-degenerative NP (Pfirmann I); mild and severe: mildly (Pfirrmann II-III) or severely (Pfirrmann IV-V) degenerative NP.

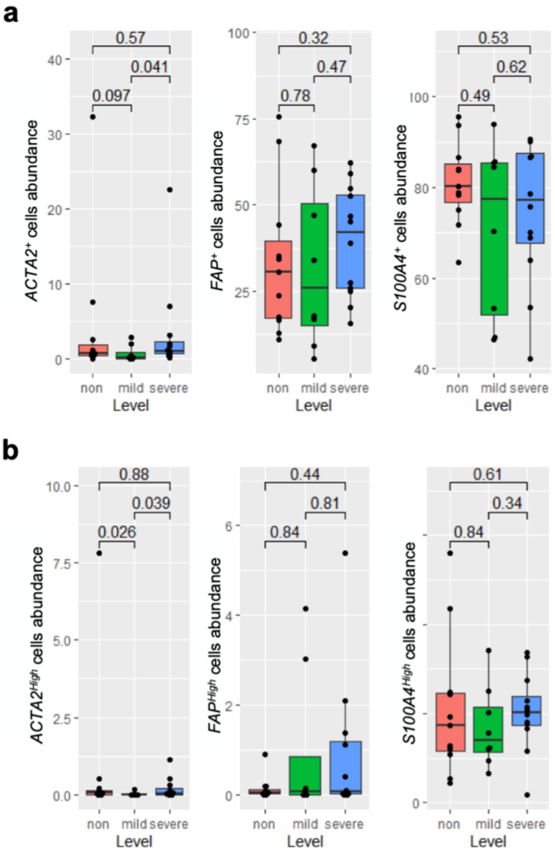

Supplementary Table S1. Summary of cell populations and markers reported in published human disc/NP tissues single-cell RNA sequencing studies.

Ref	Annotation	Defining markers	Remarks
7	C1 chonNPC	*MMP3, PDE4B, CXCL8, TNFRSF11B, MGP, ADPRHL1, FGF2, BMP2, C11orf96, SOD2, CXCL2, GPX3, CLU*	Inflammatory process
	C2 chonNPC	*PRG4, SPARC, MSMO1, CYTL1, COL2A1, ABI3BP, S100A2, VCAN, TIMP1, HAPLN1*	Cholesterol biosynthetic process and maintenance of the structure
	C3 chonNPC	*COL12A1, SAA1, MMP13, TNC, CXCL1, CHI3L2, MMP2, AEBP1, GREM1, IFITM1*	Inflammatory responses and matrix disassembly
	C4 chonNPC	*POSTN, COL1A1, COL3A1, COL1A2, SPP1, TMSB4X, LGALS1, MMP13, COL6A3, MMP14*	Fibrous characteristics
	Cartilage Progenitor	*STMN1, HIST1H4C, LGALS1, NUSAP1, TOP2A, HIST1H1B, TUBB, CKAP2, PTTG1, POSTN, CENPF, BIRC5*	NP-derived progenitor
	Fibrochondrocyte Progenitor	*MYLK, TRIB3, TCEA1, ATF3, ATF5, SYNE1, GOT1, GDF15, CYR61, DEPP1, CANX*	Protein folding quality control and ER stress
	Homeostatic chondrocytes	*FOS, JUN, ZFP36, RGS16, IER2, ID3, JUNB, ATF3, EGR1, IRF1*	Responsive to stress
	Endothelial cells	*HES1, CALCRL, COL4A1, SPARCL1, PECAM1, IFI27, PLVAP, AQP1, COL15A1, LAMA4, CD34*	
	Macrophage	*CCL3L1, CCL4L2, CCL4, IL1B, CD74, CXCL3, CXCL8, HLA-DRA, CCL3, TNF, LAPTM, TYROBP*	
14	NP progenitor	*PLA2G2A, FBLN1, SERPINF1, CFD, PTGDS, WISP2, IGFBP6, GSN, MMP2, C1R, PDGFRA, PAX1, ANGPT1, PRG4, PRRX1*	Bone morphogenesis, connective tissue development
	Stromal cells	*ACTA2, MYL9, RGS5, MYH11, SYNPO2, STEAP4, NOTCH3, MCAM, ADIRF, ADIRF, FOXC2, GJA1, HES4*	
	Stromal cells-Fibroblast	*CEMIP, AKR1C1, MGP, COMP, DNER, MELTF*	
	Stromal cells-Neurogenic	*SOX2, NGFR, NCMAP, CLDN19*	
	Stromal cells-Osteogenic	*RUNX2, DLX5, SP7, BGLAP, MMP11*	
	Pericyte	*ITGB1, CARMN, ID3, C1QTNF1, MYL6, MAP3K20, TNS1, LGI4, ABCC9, ACTA2, TAGLN, MCAM*	

	Chon1 (C1/2)	*CYTL1, IBSP, CRYAB, APOD, IL17B, RBP4, C2orf40, CHAD, SPP1, HSPA6*	Regulatory chondrocytes secreting growth factors, AF and CEP chondrocytes
	Chon2 (C3/4)	*NEAT1, MALAT1, DST, COL11A1, COL2A1, FN1, ACAN, FMOD, COMP, SLC5A3, CCNL1, WSB1*	Homeostatic chondrocytes, ECM homeostasis and circadian rhythm
	Chon3 (C5/6)	*CHRDL2, CAPS, NDUFA4L2, ABI3BP, CNMD, CHI3L1, MT1E, MT1X, VCAN, CRISPLD1, PRG4, KLF2, COL5A1, EPYC*	Effector chondrocytes, metabolic active
	Notochord NPC	*KRT19, CA3, EPYC, ENPP2, CD24, CTHRC1, ACTC1, PCSK2, RAPGEF5, RAB3B, KRT8, TBXT*	
	Endothelial cells	*CD74, IFI27, PECAM1, RAMP2, GNG11, CALCRL, VWF, EGFL7, STC1, HLA-E, CD34, CDH5, ERG*	
	Blood cells	*LYZ, S100A9, S100A8, CXCL8, SRGN, AC020656.1, MPO, CCL3, TYROBP, AZU1*	
12	Effector NPC	*MSMO1, HMGCS1, INSIG1*	Metabolic process, Positive regulation of ECM assembly
	Hypertrophic chonNPC	*FRZB, DKK1, EGR1*	Programmed cell death; ECM disassembly
	Adhesion NPC	*FN1, CRTAC1, FMOD*	Cell migration, cell–matrix adhesion.
	FibroNPC	*COL1A1, COL3A1, MMP2, COL6A1, TGFB*	Fibrosis related
	Homeostatic NPC	*RPS29, RPS21, RPL31*	Cellular homeostasis
	Regulatory NPC	*CHI3L1, NFKB, CXCL2, CXCL3, IL6*	Cellular responses to inflammation and endogenous stimuli
	NK cells	*CD94*	
	Macrophage	*CD163*	
	T cell	*TRAC*	
	G-GMP	*MS4A3, MPO, ELANE*	
	Neutrophil	*FCGR3B, HLADR*	
	G-MDSC	*ITGAM, OLR1, ARG1, CD45*	Immunopression, ROS production
11	Endothelial progenitor	*PECAM1, CDH5, CD34, KDR*	
	Erythrocytes	*HBA1, HBB*	
	Effector NP cells, EffectorNP	*COL2A1, SPARC, CLEC3A, COL3A1, FMOD, CYTL1*	ECM organization, cartilage development

	Homeostasis NP cells, HomNP	*MT1G, EMP1, HSPB1, S100A2, RGCC, ANXA1*	Responsive to stress, detoxification of inorganic compound
	Hypertrophic NP cells, HTNP	*MMP3, CXCL2, SOD2, C11orf96, BMP2, SERPINE2*	Cellular response to external stimulus, inflammatory reaction
	NP progenitor cells, NPPC	*TMSB4X, NEAT1, XIST, MALAT1*	Chondrocyte differentiation, extracellular structure organization, ossification
	CD24+ progenitor	*CD24, KRT19, KRT8, GJA1, LGALS3, APOE, CA3*	Protein synthesis, cellular pluripotency
	MK167+ progenitor	*MK167, CENPF, STMN1, POSTN, TUBB, TUBA1B, COL1A1*	Epithelial-mesenchymal transition (EMT), inflammatory response
	AF cells	*COL1A1, CRTAC1, ASPN, MMP2*	

Supplementary Table S2. Abundance of fibrocyte in cell clusters. Number of $ITGAM^{+}COL1A1^{+}$ and $PTPRC^{+}COL1A1^{+}$ cells in each cell cluster (as described in **Fig. 3**) were counted and compared to the count of fibrocytes.

**Cell cluster**	***ITGAM⁺COL1A1⁺***		***PTPRC⁺COL1A1⁺***		***Fibrocytes***	
	**Count**	**%**	**Count**	**%**	**Count**	**%**
ChonNP	38	3.47	54	3.11	89	4.16
FibroNP	97	8.87	69	3.97	160	7.48
RegNP	36	3.29	30	1.73	64	2.99
ProgNP	0	0.00	3	0.17	3	0.14
CyclingNP	4	0.37	6	0.35	9	0.42
Neutrophil	411	37.57	647	37.23	732	34.24
G-MDSC	21	1.92	52	2.99	63	2.95
Macrophage	462	42.23	629	36.19	756	35.36
T cell	16	1.46	238	13.69	243	11.37
Endothelial cell	4	0.37	3	0.17	7	0.33
Pericyte	4	0.37	6	0.35	10	0.47
Erythrocyte	1	0.09	1	0.06	2	0.09

Supplementary Table S3. Safety and efficacy evaluation of diptheria toxin injection in CD11b-DTR mice. Injection was performed at indicated dosage for 1-3 times within week 1-5. At week 8, number of CD11b+ cells in peripheral blood was measured by flow cytometry and depletion efficiency was calculated. Three mice were tested for each condition. A: >1 living mice at week 8; D: all mice died by week 8.

**Dosage**	**Frequency**	**Time (week)**					**Depletion efficiency**
		**1**	**3**	**5**		**8**	
5ng/g	3	√	√	√		A	55.80%
	2	√		√		A	45.78%
10ng/g	3	√	√	√		D	N/A
	2	√		√		A	65%
15ng/g	2	√		√		D	N/A
	1	√				A	55.46%
20ng/g	2	√		√		D	N/A
	1	√				A	68.50%
25ng/g	2	√		√		D	N/A
	1	√				D	N/A

Supplementary Table S4. Demographics of IVD donors. Disc samples were harvested from AIS subjects undergoing deformity correction and DDD subjects undergoing disc excision and spinal fusion. Grade of disc degeneration was determined by MRI according to Pfirrmann Scale (I-V). F, female; M, male; AIS, adolescent idiopathic scoliosis; DDD, degenerative disc disease; ND, not determined.

Sample ID	Gender	Age (Years)	Level	Pathology	Disc grade
1	F	13	L2-3	AIS	ND
2	F	13	L2-3	AIS	ND
3	M	15	L2-3	AIS	ND
4	F	15	L2-3	AIS	ND
5	F	16	L1-2	AIS	ND
6	F	15	L2-3	AIS	ND
7	M	28	L5-S1	DDD	IV
8	M	40	L3-L4	DDD	III
9	F	42	L4-L5	DDD	IV
10	F	47	L3-L4	DDD	V
11	F	56	L4-L5	DDD	III
12	M	58	L3-L4	DDD	V
13	M	59	L4-L5	DDD	IV
14	F	45	L4-L5	DDD	IV
15	M	71	L4-L5	DDD	IV

Supplementary Table S5. Antibodies used for immunofluorescence and flow cytometry (FC) analysis.

Product Cat#	Antigen	Host	Conjugation	Application
EPR14664	Aggrecan	Rabbit	N/A	ACAN in human NP (Supplementary Fig. S3)
Ab34710	Collagen I	Rabbit	N/A	COL1 in human NP (Supplementary Fig. S3); FC for COL1 (Supplementary Fig. S5); Co-stain with CD34 in Cd11b-DTR mice IVDs
Ab88147	Collagen I	Mouse	N/A	FC for COL1 (Fig. 3); Co-stain with CD45, αSMA in human NP (Fig. 3)
EPR12268	Collagen II	Rabbit	N/A	COL2 in human NP (Supplementary Fig. S3)
Ab7778	Collagen III	Rabbit	N/A	COL3 in human NP (Supplementary Fig. S3)
EPR5368	αSMA	Rabbit	N/A	FC for αSMA (Fig. 2); αSMA in human NP (Fig. 2); co-stain with GFP in CD11b-DTR mice IVDs (Fig. 4&5)
Ab7817	αSMA	Mouse	N/A	Co-stain with CD45 in human NP (Fig. 3)
EPR20021	Fap-α	Rabbit	N/A	FC for FAPα (Fig. 2); FAPα in human NP (Fig. 2);
EPR2761-2	Fsp1	Rabbit	N/A	FC for FSP1 (Fig. 2); FSP1 in human NP (Fig. 2);
Ab23910	CD45	Rat	N/A	Co-stain with COL1 and αSMA in CD11b-DTR mice IVDs (Fig. 5)
Ab10558	CD45	Rabbit	N/A	Co-stain with COL1 in human NP (Fig. 3), and GFP in CD11b-DTR mice IVDs (Fig. 4)
9B10D4	CD34	Mouse	N/A	Co-stain with COL1 and αSMA in human NP (Supplementary Fig. S8)
Ab184308	CD11b	Rabbit	N/A	Co-stain of CD11b with GFP in CD11b-DTR mice IVDs (Fig. 4); FC for CD11b in CD11b-DTR mice IVDs (Supplementary Fig. S12)
Ab5450	GFP	Goat	N/A	GFP in CD11b-DTR mice IVDs (Fig. 4)
Ab150077	Rabbit IgG H&L	Goat	Alexa Fluor® 488	Immunostaining of COL1/3, ACAN, αSMA, FAPα (Supplementary Fig. S3 and Fig. 2); FC for αSMA, FAPα, FSP1, COL1 and CD45 in human NP cells (Fig. 2& 3, Supplementary Fig. S5); co-stain of CD45 with COL1 and αSMA in human NP (Fig. 3); co-stain of COL1 with CD45 in CD11b-DTR mice (Fig. 5b)
Ab150080	Rabbit IgG H&L	Goat	Alexa Fluor® 594	Immunostaining of COL2, FSP1 in human NP (Supplementary Fig. S3 & Fig. 2) and COL1 in CD11b-DTR mice IVDs (Fig. 5); FC for αSMA, Fap-α and Fsp1 (Fig. 2); Co-stain of CD45 with COL1 (Fig. 3), COL1 and αSMA

				with CD34 in human NP (Supplementary Figu. S8); co-stain of αSMA with CD45 in CD11b-DTR mice (Fig. 5c)
Ab150116	Mouse IgG H&L	Goat	Alexa Fluor® 594	FC for COL1 (Fig. 3); Co-stain of COL1 and αSMA with CD45 in human NP (Fig. 3)
Ab150160	Rat IgG H&L	Goat	Alexa Fluor® 594	Co-stain of CD45 with COL1 in CD11b-DTR mice IVDs (Fig. 5b)
Ab150157	Rat IgG H&L	Goat	Alexa Fluor® 488	Co-stain of CD45 with αSMA in CD11b-DTR mice IVDs (Fig. 5c)
Ab150113	Mouse IgG H&L	Goat	Alexa Fluor® 488	Co-stain of COL1 and αSMA with CD34 in human NP (Supplementary Fig. S8)
Ab150129	Goat IgG H&L	Donkey	Alexa Fluor® 488	GFP and co-stain with CD11b, CD45, αSMA in CD11b-DTR mice IVDs (Fig. 4)
Ab175470	Rabbit IgG H&L	Donkey	Alexa Fluor® 568	Co-stain of CD11b, CD45, αSMA with GFP in CD11b-DTR mice IVDs (Fig. 4)

Supplementary Table S6. Primers for real-time PCR.

Gene name	Forward (5' to 3')	Reverse (5' to 3')
ACTA2	CTATGAGGGCTATGCCTTGCC	GCTCAGCAGTAGTAACGAAGGA
FAP	ATGAGCTTCCTCGTCCAATTCA	AGACCACCAGAGAGCATATTTTG
S100A4	GATGAGCAACTTGGACAGCAA	CTGGGCTGCTTATCTGGGAAG
COL1A1	ATCAACCGGAGGAATTTCCGT	CACCAGGACGACCAGGTTTTC
COL3A1	GCCAAATATGTGTCTGTGACTCA	GGGCGAGTAGGAGCAGTTG
COL2A1	TGGACGATCAGGCGAAACC	GCTGCGGATGCTCTCAATCT
ACAN	ACTCTGGGTTTTCGTGACTCT	ACACTCAGCGAGTTGTCATGG
GAPDH	GGAGCGAGATCCCTCCAAAAT	GGCTGTTGTCATACTTCTCATGG

169 **Supplementary Table S7. Description of genes symbols.**

**Gene acronyms**	**Full name**
ACAN	Aggrecan
ACTA2	Smooth Muscle Actin, alpha 1
ALDOA	Aldolase A
ANGPTL4	Angiopoietin-Like Protein 4
ASPN	Asporin
CAPZA1	Capping Protein (Actin Filament) Muscle Z-line, alpha 1
CCR1	C-C Chemokine Receptor Type 1
CD163	CD163
CD34	CD34
CD68	CD68
CHI3L1	Chitinase-3-Like Protein 1
CHI3L2	Chitinase-3-Like Protein 2
CNMD	Chondromodulin
COL1A1	Collagen, type I, alpha 1
COL2A1	Collagen, type II, alpha 1
COL3A1	Collagen, type III, alpha 1
CXCR4	C-X-C Chemokine Receptor Type 4
FAP	Fibroblast Activation Protein
GNB1	Guanine Nucleotide-Binding protein subunit beta-1
GPX3	Glutathione Peroxidase 3
HBB	Hemoglobin, beta
HLA-DRA	Human Leukocyte Antigen, DRA
HTRA1	High-Temperature Requirement Protein A1
ITGAM	Integrin alpha M
ITGB1	Integrin, beta 1
LGALS1	Lectin, Galactoside-Binding, Soluble, 1
LGALS3BP	Lectin, Galactoside-Binding, Soluble, 3 Binding Protein
LYZ	Lysozyme
MMP3	Matrix metallopeptidase 3
MPO	Myeloperoxidase
PDGFRA	Platelet-derived growth factor receptor, alpha polypeptide
PECAM1	Platelet Endothelial Cell Adhesion Molecule 1
PL2G2A	Phospholipase A2, Group IIA
POSTN	Periostin
PTPRC	Protein Tyrosine Phosphatase, Receptor Type, C
RGS5	Regulator of G-protein Signaling 5

RORA	RAR-Related Orphan Receptor A
S100A4	S100 Calcium Binding Protein A4
S100A9	S100 Calcium Binding Protein A9
SOX9	SRY-Related High Mobility Group-Box Gene 9
SPP1	Secreted Phosphoprotein 1
STMN1	Stathmin 1
TAGLN	Transgelin
TGFBI	Transforming Growth Factor, Beta-Induced
TOP2A	Topoisomerase (DNA) II Alpha
TRAC	T Cell Receptor Alpha Constant
TRBC2	T cell receptor beta constant 2
VIM	Vimentin

Supplementary methods

Disc height index calculation

Based on the X-rays for coccygeal IVDs of Cd11b-DTR mice, the lengths of the discs and adjacent vertebral bodies were measured with Image J (Version 1.42, National Institutes of Health). Disc height index (DHI) was calculated according to the formula: DHI = the disc height (averaging three measurements of the heights of lateral and middle portions of the disc)/ the vertebral body height (averaging three measurements of the heights of the lateral and middle adjacent caudal vertebral body), and. Level C6/7 of tail disc was un-operated and used as reference for DHI calculation.
